# Grassland allergenicity increases with urbanisation and plant invasions

**DOI:** 10.1007/s13280-022-01741-z

**Published:** 2022-05-20

**Authors:** Maud Bernard-Verdier, Birgit Seitz, Sascha Buchholz, Ingo Kowarik, Sara Lasunción Mejía, Jonathan M. Jeschke

**Affiliations:** 1 Institute of Biology, Freie Universität Berlin, Königin-Luise-Straße 1-3, 14195 Berlin, Germany; 2 Berlin-Brandenburg Institute of Advanced Biodiversity Research (BBIB), Berlin, Germany; 3 Leibniz Institute of Freshwater Ecology and Inland Fisheries (IGB), Müggelseedamm 301, 12587, Berlin, Germany; 4 Department of Ecology, Technische Universität Berlin, Rothenburgstraße 12, 12165 Berlin, Germany; 5 Institute of Landscape Ecology, University of Münster, Heisenbergstraße 2, 48149 Munster, Germany; 6 Faculty of Biology, University of Barcelona, Barcelona, Spain

**Keywords:** Alien plants, Biochemical diversity, Ecosystem disservices, Novel ecosystems, Public health, Urban ecology

## Abstract

Pollen allergies have been on the rise in cities, where anthropogenic disturbances, warmer climate and introduced species are shaping novel urban ecosystems. Yet, the allergenic potential of these urban ecosystems, in particular spontaneous vegetation outside parks and gardens, remains poorly known. We quantified the allergenic properties of 56 dry grasslands along a double gradient of urbanisation and plant invasion in Berlin (Germany). 30% of grassland species were classified as allergenic, most of them being natives. Urbanisation was associated with an increase in abundance and diversity of pollen allergens, mainly driven by an increase in allergenic non-native plants. While not inherently more allergenic than native plants, the pool of non-natives contributed a larger biochemical diversity of allergens and flowered later than natives, creating a broader potential spectrum of allergy. Managing novel risks to urban public health will involve not only targeted action on allergenic non-natives, but also policies at the habitat scale favouring plant community assembly of a diverse, low-allergenicity vegetation. Similar approaches could be easily replicated in other cities to provide a broad quantification and mapping of urban allergy risks and drivers.

**Supplementary Information:**

The online version contains supplementary material available at 10.1007/s13280-022-01741-z.

## Introduction

Pollen allergies are a leading cause of chronic respiratory diseases worldwide, affecting up to 40% of the population (Tong and Lin 2015) and have been on the rise for several decades as a result of multiple global changes (D’Amato et al. 2007; Linneberg et al. 2007). Increasingly urban lifestyles, projected to reach 68% of the global population by 2050 (United Nations 2018), appear as a major driver of this trend (von Mutius 2016). In addition, rising CO_2_ levels and warmer climates increase the quantity of pollen released into the atmosphere (Ziska and Beggs 2012; Barnes 2018), while land-use changes and biological invasions are modifying plant species composition, thus, altering the spatio-temporal composition of airborne pollens (D’Amato et al. 2007; Grewling et al. 2015). Nowhere do these global changes overlap more than in cities, and nowhere are they more likely to affect human populations.

The prevalence of pollen allergenic rhinitis in urbanised areas is higher than in rural areas, and has been rising faster than anywhere else (D’Amato et al. 2007; Ziska and Beggs 2012). This appears to be partly due to a lack of exposure during lifetime to a diversity of microbes and other allergens (i.e. the *biodiversity* or *hygiene hypothesis*; Hanski et al. 2012, Sandifer et al. 2015). Studies have shown that children growing up in allergen-rich environments—including animals, microbes, house dust mites and pollens—such as farms (Riedler et al. 2001) or near green areas (Ruokolainen et al. 2015; Paciência et al. 2021) are less prone to developing allergies. Air pollution is also a factor increasing allergy prevalence in cities, via multiple mechanisms affecting both lung sensitivity and the allergenicity of pollen particles (Nicolaou et al. 2005; Reinmuth-Selzle et al. 2017). Finally, urbanisation is associated with an increase in temperatures, the so-called *urban heat-island effect*, which extends the duration of the growing season in temperate and boreal climates (Ziska et al. 2003). Earlier and longer flowering in cities extends the allergy season (Jochner and Menzel 2015), while higher temperatures and increased CO_2_ levels can result in increased pollen production (Ziska et al. 2003).

Urbanisation affects not only the quantity but also the quality and diversity of pollen allergens released in the air. The taxonomic composition of allergenic airborne pollen changes between sub-urban areas and the city centre, reflecting changes in vegetation composition (Rodríguez-Rajo et al. 2010; Bosch-Cano et al. 2011; Grewling et al. 2015; Brennan et al. 2019). Actively managed parts of the urban vegetation, including trees and ornamentals in streets and gardens, can pose major allergenic risks (Cariñanos and Casares-Porcel 2011). In unmanaged urban vegetation ruled by community assembly dynamics, we expect the composition of allergens to change with the turnover of plant species from rural to urban plant communities, as plants unable to cope with urban disturbances are replaced by plants able to survive or even thrive in urban habitats (Kowarik 1990).

Urban floras have more non-native species than semi-natural or rural areas (Kowarik 1990; Pyšek 1998), as a result of both high rates of introduction—intentionally or not (Saul et al. 2017)—and high rates of disturbance (Kowarik 1990). Plant invasions in cities typically introduce ecological novelty (Heger et al. 2019), as species with different biogeographic origin and eco-evolutionary experiences (sensu Saul et al. 2013) may have unique traits. Consequently, non-native species may potentially produce new pollen allergens absent from the native flora (Potgieter et al. 2017), or occupy different phenological niches (Godoy et al. 2009; Wolkovich and Cleland 2014), thereby modifying the allergy season. Some non-native plants are indeed notable allergens—for instance, non-native ornamental trees introduced for urban greening (Cariñanos and Casares-Porcel 2011), or invasive ragweed in Europe (*Ambrosia artemisiifolia*; Essl et al. 2015)—but we know generally very little about the potential allergy risk posed by the pool of non-native urban plants, and whether it differs from natives.

Allergy risk in cities is most often monitored by quantifying airborne pollen loads using traps, but these cannot locate the precise plant assemblages producing the allergens (Werchan et al. 2017) nor tell us even whether the different allergenic species coexist in a plant community (Rodríguez-Rajo et al. 2010; Katz et al. 2019). To characterise pollen emission sources and identify the drivers of their spatial distribution, allergenic vegetation must be mapped at ground level. While previous urban studies covered mostly allergy risks of trees and shrubs (Cariñanos et al. 2016, 2017; Kasprzyk et al. 2019; Velasco-Jiménez et al. 2020; Aerts et al. 2021), herbaceous vegetation has received less attention (but see Hruska 2003), in particular outside parks or in non-managed areas, and never along a well-defined urbanisation gradient.

Grassland is a common and diverse ecosystem type in cities (Fischer et al. 2013b) and a potential large source of allergens, with grasses and weeds often ranking as high as urban trees in pollen production and allergenicity (D’Amato et al. 2007). While urbanisation and plant invasion have been shown to modulate urban grassland composition and functioning (e.g. Onandia et al. 2019), consequences of these drivers on the diversity and abundance of allergens are unknown. We explored how the turnover in grassland plant species composition and abundance in response to increasing urbanisation and plant invasion may transform the allergenic pollen environment for human residents.

Our aim was to quantify the changes in allergenic potential of urban grasslands along a double gradient of urbanisation and plant invasion. We used vegetation data recorded in 2017 on 56 plots of extensively managed dry permanent grasslands in Berlin, Germany. We gathered available online information on plant allergenicity, allergenic pollen molecules and flowering phenology for all 216 herbaceous plant species observed in the plots. We used an approach at the plant community level to characterise the allergenic potential of grasslands, and test how it gradually changes from rural to urban areas. We addressed the following research questions:

1. Does the abundance and/or diversity of allergens in grasslands increase with urbanisation?
2. What is the contribution of non-native species to the allergenic potential and biochemical diversity of urban grasslands?
3. Is timing of the allergy season modified by urbanisation and plant invasions in grasslands?

We expected highly invaded and more urban grassland communities to have a higher abundance and diversity of allergens, as well as a longer flowering season, than less urban and less invaded grasslands.

## Materials and Methods

### Study area

**Fig. 1:**
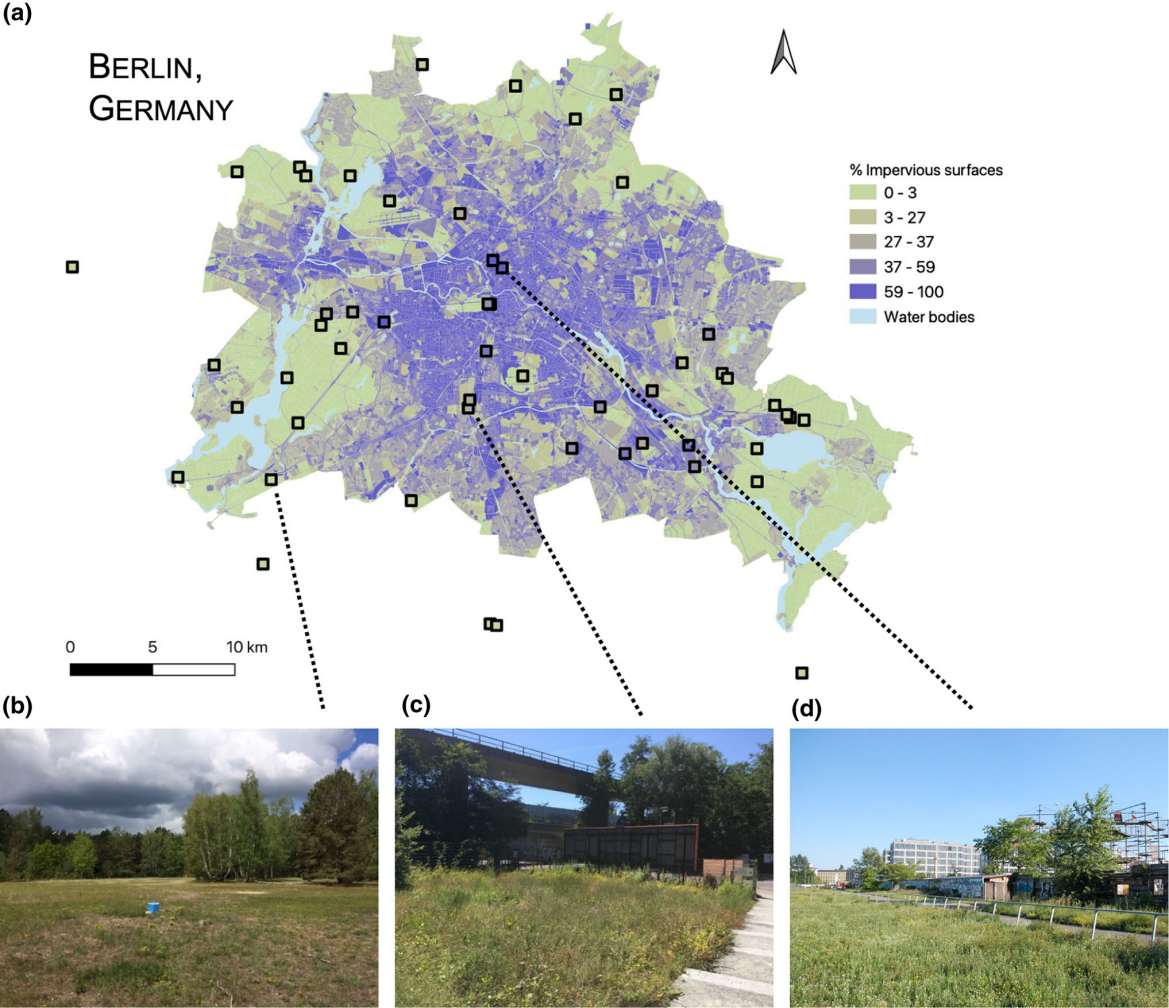
**a** Map of 56 dry grassland plots in the city of Berlin, Germany. Each square corresponds to a 4 × 4 m grassland plot. The color gradient represents the percentage of impervious surfaces (data from the Berlin senate). Note that five plots were located outside the borders of Berlin, in rural areas of the Brandenburg region. Example grassland plots: **b** rural grassland in a wide clearing of the Wannsee forest; **c** sub-urban grassland patch in the Schöneberg district; **d** urban grassland next to Nordbahnhof train station. Credit: Technische Universität Berlin 2016

Our study focused on dry grassland in Berlin (Germany), an anthropogenous type of vegetation that is usually lightly managed by mowing once or twice a year—without irrigation, fertilisation, herbicide application nor intensive trampling (von der Lippe et al. 2020). Dry grasslands can harbour a high plant diversity and are frequent around the city, in roadside green spaces, large parks, former airfields and many urban land-use types (Fischer et al. 2013b). In 2016, 56 grassland plots (4 × 4 m) were selected across the city to cover a wide gradient of urbanisation (Fig. 1). These field sites were part of a collaborative research platform called the *CityScapeLab Berlin*, created as part of the *Bridging in Biodiversity Science* project*, and* spanned a wide range of habitats, from rural landscapes at the urban fringe to highly urbanised settings along road median strips or railroad tracks. They represent a standardised model ecosystem with reduced environmental heterogeneity since all these dry grasslands could be assigned to the same vegetation type (i.e. Sedo-Scleranthetea), following the phytosociological vegetation classification of Braun-Blanquet (see von der Lippe et al. 2020 for further details).

### Vegetation surveys

Vegetation surveys were carried out in spring and summer 2017, recording per cent cover of all vascular plant species following the nomenclature by Rothmaler (Jäger 2017; details in von der Lippe et al. 2020). To improve species matches with allergen databases, possible species synonyms were later extracted using the function *iplant_resolve* from the R package *taxize* (Chamberlain and Szöcs 2013) using the *Tropicos* database as reference (http://urbanis.tropicos.org, accessed 23 Nov 2019). Taxonomic classification from family to sub-tribe was extracted from the NCBI database using the function *tax_name* from the same package. In total, 234 vascular plant species were recorded across the 56 sites. From these 234, we excluded the occasional tree individuals recorded as seedlings and saplings in the field layer, choosing to focus instead on the main herbaceous layer of the vegetation. This brought the total number of plant species to 216, spanning 123 genera and 37 families. We collected information on the introduction status of plant species in Germany, and specifically the Berlin area, from Seitz et al. (2012), distinguishing native species (69% of species) from archaeophytes (i.e. non-natives introduced before 1492 CE; 17%) and neophytes (i.e. non-natives introduced since 1492 CE; 14%). The BiolFlor database (Biological and ecological traits of the flora of Germany; Klotz et al. 2002) provided broad estimates of species flowering phenology (at the month level) for Germany. Mean differences in species flowering dates between the three groups of species were tested using bootstrapped non-parametric ANOVAs based on kernel regressions (R package *np*).

### Environmental gradients

We quantified urbanisation levels based on the percentage of impervious surfaces in a 500 m buffer around the grassland patches (details in von der Lippe et al. 2020), a commonly used proxy for urbanisation (McKinney 2002). Impervious surfaces, i.e. sealing of the soil by buildings or roads, is a characteristic feature of the urban environment which correlates with other aspects of urbanisation, such as a warmer microclimate, reduced evapotranspiration or reduced rain infiltration (Endlicher et al. 2011). Human population density in a 500 m buffer was tightly correlated to the percentage of impervious surfaces in our study system (Spearman’s rank correlation: *ρ* = 0.88, *n* = 56, *P* < 0.0001).

We also investigated a gradient of biological invasion, quantified by the proportion of neophyte species in each grassland patch. As previously shown in our study system (Schittko et al. 2020; though we use here a subset of species excluding tree seedlings), the more urban grasslands (higher % impervious surfaces) tended to have a higher proportion of neophytes (*ρ* = 0.46, *n* = 56, *P* < 0.0001), but the correlation remained relatively loose: some highly urban areas still had a low level of neophyte invasion, and some rural sites were highly invaded. This allowed us to potentially disentangle the gradients of urbanisation and plant invasion.

### Species allergenicity and allergen molecules

We collected data on the allergenic properties of all 216 plant species from multiple public online allergen databases (cf. details on data collection in Appendix S3). These databases record information on the identity of allergen molecules present in the pollen of plant species, sometimes accompanied by a scoring of allergenic severity, information on known symptoms and prevalence in human populations (mostly from the Northern hemisphere). Allergen molecules are peptides belonging to a small range of protein families (Radauer and Breiteneder 2006), and often a single species will produce several allergen peptides in its pollen. Any plant species listed in these databases as having at least one allergenic pollen molecule was classified as allergenic. This list of allergenic species was limited by the current state of knowledge in allergy research: the absence of a species in the database may be due either to a real absence of allergenic potential or a lack of research on this species. To fill this publication gap, and based on strong evidence of cross-reactivity of allergens within closely related species (Weber 2004; Radauer and Breiteneder 2006; D’Amato et al. 2007), we expanded the definition of allergenicity to include all congenerics of known allergenic species, as well as all grass species. In total, we classified 74 species as allergenic out of 216. Similarly, extrapolating allergen molecules to the genus level (or tribe level for grasses) allowed us to assign 122 unique allergen molecules to 53 out of the 74 allergenic species.

### Species potential allergenic values

We calculated the potential allergenic value (PAV) of each species following the method proposed by Cariñanos et al. (2016). We assigned an overall allergenic potential score to species by multiplying three main factors: *allergenicity score* (from 0 to 4; cf. details in Online Appendix S3); *pollination syndrome* (four categories: 0—no pollen emissions, 1—biotic pollination or low emissions, 2—mixed pollination with moderate pollen emissions and 3—wind-pollinated with high pollen emissions) and *phenology score*, based on the duration of flowering (scores from 1 to 3: < 1 month, < 2 months and > 2 months). These ordinal scores were multiplied, establishing a species score ranging from 0 to 36.

### Community allergenicity

We characterised the allergenic potential of each grassland community (i.e. each grassland plot) in four different ways, based on (1) species presence and cover; (2) species PAV; (3) allergenic molecules produced and (4) flowering phenology. We repeated all calculations by introduction status, i.e. for the subsets of native, archaeophyte and neophyte species.

We first calculated the number of allergenic species present in each community (*allergenic species richness*) and the proportion they represent (*allergenic species proportion*) as well as their cumulative per cent cover in the plot (*allergenic cover*). Second, we calculated the mean allergenic potential value of each plant community, both unweighted (*mean*_*PAV*_) and weighted by species cover (community weighted mean: CWM_PAV_). Note that the latter calculation is equivalent to the often-used iUGZA (Index of Urban Green Zone Allergenicity; Cariñanos et al. 2014), but neglects differences in heights and crown sizes between species, these being less relevant for herbaceous communities, and does not provide a ratio to a hypothetical maximum, which here is necessarily always 36 (maximum PAV). Third, we calculated the diversity of allergen molecules in each grassland community as the total number of unique allergen molecules potentially produced by plant species present in the community (*allergen richness*)*.* Allergen diversity was also characterised at the biochemical level by counting unique protein families (*allergen family richness*). Finally, we used information on species flowering phenology to estimate the average number of allergenic species potentially flowering simultaneously per month in each plot (*mean monthly allergenic richness*). This last approach was also used to calculate mean monthly allergen molecule and allergen family richness.

### Statistical analyses

We investigated the variations in community allergenicity metrics along the environmental gradients using generalised linear models fitted individually for each predictor. We tested two main environmental gradients: (1) urbanisation, captured by the percentage of impervious surfaces in a 500 m buffer around the plot and (2) plant invasion, captured by the proportion of neophyte species in the grassland community. Based on preliminary observation of model residuals, species count data and allergenic family richness were modelled with Poisson distributions, allergen molecule richness with Negative Binomial distributions and species proportions with Quasi-binomial distributions (adjusted for overdispersion; R packages ‘lme4’ and ‘MASS’). In addition, we explored possible interactions between the two gradients by applying model selection across all combinations of predictors based on AIC (R package MuMin; Bartoń 2020). The “best model” selected had the lowest AIC_c_, so long as it was better than the null model (ΔAIC_c_ < 2). Finally, we quantified turnover in allergen composition between grassland communities using Jaccard’s dissimilarity index to quantify changes in occurrence of allergenic species, allergen molecules and allergen protein families. Using distance-based redundancy analyses (R package “vegan”, function “dbRDA”; Oksanen et al. 2016), we quantified possible trends in turnover with urbanisation (% Impervious surfaces) and proportion of neophytes. We also tested Bray–Curtis dissimilarities taking species cover into account, but these are not shown as they had similar trends.

To test for possible biases due to spatial autocorrelation, we also calculated Moran’s index on model residuals and tested it against a null expectation (Moran.I, R package “ape”; Paradis and Schliep 2019). All analyses were performed in R (version 4.0.3) and can be reproduced from the github repository (data and R code: https://github.com/maudbv/UrbanGrasslandAllergens; https://doi.org/10.5281/zenodo.4740338).

## Results

### Species allergenicity

We identified in total 74 allergenic species (34.3% of all 216 recorded species) across the 56 grassland plots. We identified allergen molecules for 53 of these species, the number of allergen molecules per species ranging ranged from 1 to 13 (median = 6, mean = 6.58, sd = 3.2). Generally, the higher the number of allergen molecules recorded per species, the higher the allergenicity score (*as defined in* Table S3.2) of the species (Spearman rank correlation: *ρ* = 0.88, *n* = 53, *P* < 0.0001).

Pollination syndrome and taxonomic family were good predictors of species allergenicity. Wind-pollinated species, which represented 23% of all species, were 55 times more likely to be allergenic than species with other pollination modes (Fisher exact test: odds ratio = 55.07, *P* < 0.0001) and tended to have higher allergenicity scores (*G*-test: *G* = 126.94, *df* = 4, *P* < 0.0001). Allergenic species were over-represented in certain plant families (*G*-test: *G* = 168.66, *df* = 36, *P* < 0.0001), with allergenic species restricted to 10 out of 37 plant families. Allergenics were particularly over-represented in the Poaceae (36 allergenic species; representing 100% of family members by design; cf. Methods), Cyperaceae (6; 100%), Chenopodiaceae (1; 100%), Fabaceae (11; 50%), Asteraceae (11; 33%) and Plantaginaceae (3; 33%).

Allergenic species from the same taxonomic family tended to produce allergens from the same protein families (Permanova: *R*^2^ = 0.57, *F* = 10.08, *P* < 0.0001; cf. Fig. S1), although that pattern was likely exacerbated by our decision to assign common allergens at the genus or the tribe level. The highest number of allergen molecules per species tended to be in the Plantaginaceae (median = 11, max = 11), Poaceae (median = 8, max = 13) and Asteraceae (median = 2, max = 13) families.

### Allergenicity of native vs. non-native species

Allergenic species were equally frequent among neophytes (36.7%), archaeophytes (29.7%) or native species (34.9%) in our dataset (G-test: *G* = 0.45, *df* = 2, *P* = 0.7992). There was also no difference in potential allergenic values (PAV; negative binomial GLM: *R*^2^ = 0.01, *df* = 71, *P* = 0.725), in number of allergen molecules per species (Quasipoisson GLM: *R*^2^ = 0.03, *df* = 50, *P* = 0.672), nor in number of allergen families per species between these groups (Poisson GLM: *R*^2^ = 0.04, *df* = 50, *P* = 0.4496). However, neophyte allergens represented a more diverse spectrum of protein families (26 different allergen families) than the allergens contributed by archaeophytes (17) or natives (19). This was observed despite the low number of neophyte allergenic species compared to the large number of native allergenic species. Rarefaction curves confirmed that neophyte species tended to contribute more biochemically diverse allergens per species (Fig. S1A). For 10 allergenic species picked randomly, there were on average 24.75 (sd = 2.16) unique allergen families contributed by neophytes, as opposed to 12.06 (sd = 2.74) for natives or 16.81 (sd = 0.59) for archaeophytes.

### Allergenic species diversity

On average, there were 12.7 ± 3.5 allergenic species (45% of species; range: 31% to 58%) per grassland plot, occupying 57% (± 20%) cumulated vegetation cover per plot (range: 15% to 109%). Allergenic species were mostly native (on average 80.5% of allergenic species per plot; range 54.5–100%). Allergenic species richness, but not cover, tended to increase with total species richness (Spearman rank test: *ρ* = 0.84, *n* = 56, *P* < 0.001; details of pairwise diversity correlations in Table S1).

**Fig. 2:**
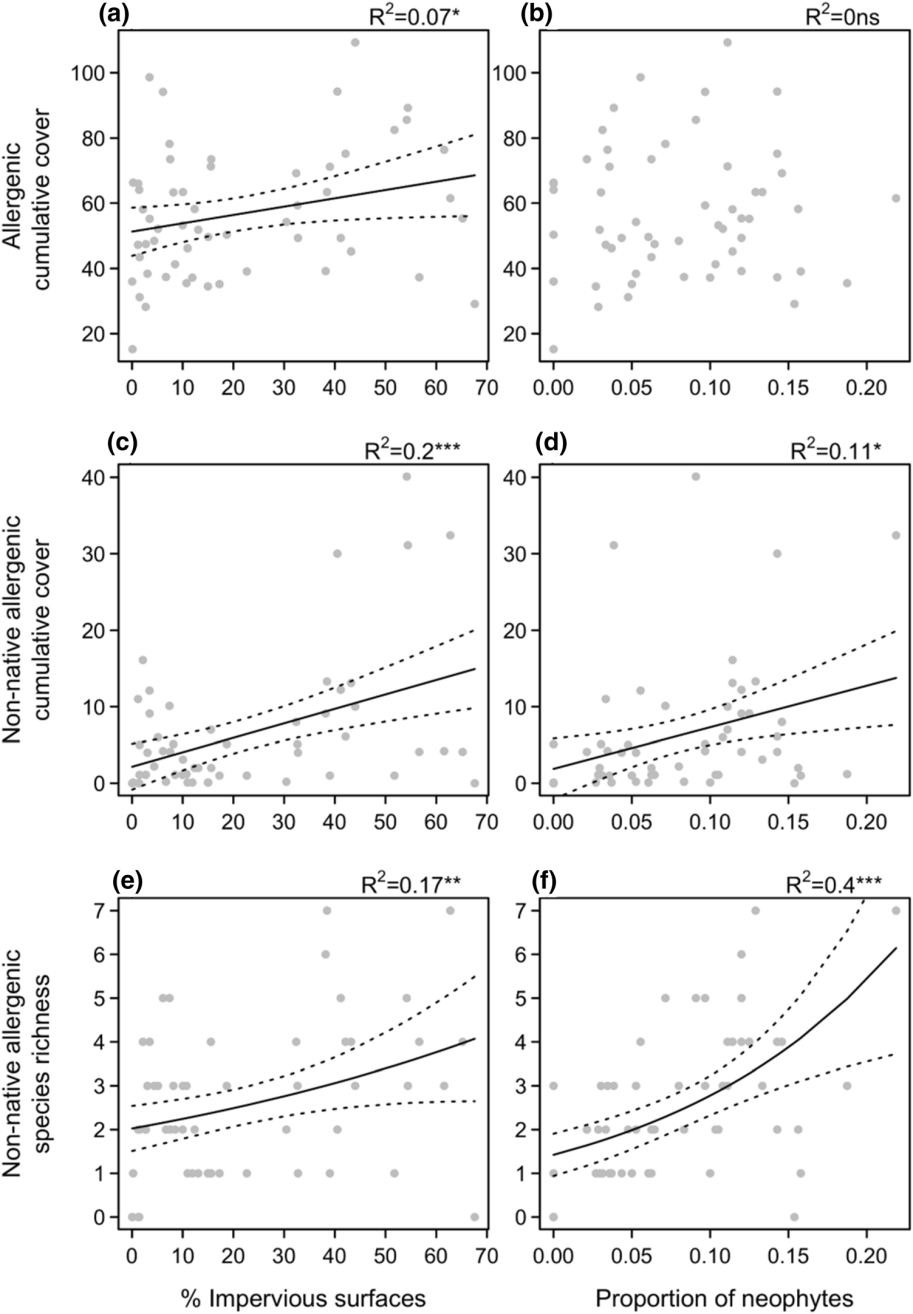
Allergenic species cover and richness in 56 Berlin grasslands along gradients of urbanisation and biotic novelty. Cumulative cover of all allergenic species (**a**, **b**), as well as the cover (**c**, **d**) and richness (**e**, **f**) of non-native allergenics are represented as a function of urbanisation (% impervious surfaces in a 500 m radius) and the proportion of neophytes in each grassland community. Gaussian and Poisson regressions were fitted for cover and richness, respectively. Regression lines with standard errors are represented when significant (solid line; *P* < 0.05). Statistics for the models are given above panels (Likelihood-ratio test: *P < 0.05; **P < 0.01; ***P < 0.001; ns, *P* ≥ 0.05)

Cumulative per cent cover of allergenics increased with urbanisation (i.e. percentage of impervious surface in a 500 m buffer; Fig. 2a; Table 1), from 46.2 ± 18.6% cover in less urban sites (< 2% impervious surfaces), to 64.6 ± 22.7% cover in the more urban ones (> 50% impervious surfaces). Total allergenic species richness showed no trends along the urbanisation gradient, nor with the proportion of neophytes (Table 1). However, we did detect a significant turnover in allergenic species composition with urbanisation (distance-based redundancy analyses; details in Appendix S4).

**Table 1:** Models for allergenic species richness, proportion, and cover as a function of urbanisation and biotic novelty. Statistics for linear models (cover), and Binomial (proportion) or Poisson (richness) generalised linear models are presented. Models were fitted for each of the three predictors independently. *R*^2^ values for GLMs are Nagelkerke pseudo-*R*^2^, and *P* values correspond to a Likelihood-ratio test compared to the null model. Significant models (*P* < 0.05) are in bold. Non-native allergenic species correspond to both allergenic neophytes and archaeophytes combined

Subset of species	Metric	*df*	% Impervious surfaces				Proportion of neophytes			
			coef	SE	*P*	*R*^2^	coef	SE	*P*	*R*^2^
Allergenic species	Richness	54	0.002	0.002	0.3865	0.021	0.684	0.708	0.3350	0.026
	Cover	54	**0.256**	**0.127**	**0.0483**	**0.070**	16.205	50.848	0.7512	0.002
Native allergenics	Richness	54	− 0.001	0.002	0.6859	0.005	− 0.916	0.803	0.2527	0.039
	Prop	54	**− 0.012**	**0.004**	**0.0064**	**0.169**	**− 8.280**	**1.534**	** < 0.0001**	**0.462**
	Cover	54	0.067	0.116	0.5651	0.006	− 38.100	44.618	0.3969	0.013
Archaeophyte allergenics	Richness	53	0.008	0.005	0.0931	0.075	**4.546**	**1.850**	**0.0149**	**0.154**
	Prop	53	0.007	0.005	0.2023	0.042	**4.550**	**1.958**	**0.0207**	**0.127**
	Cover	53	**0.122**	**0.039**	**0.0031**	**0.154**	3.967	16.099	0.8063	0.001
Neophyte allergenics	Richness	46	**0.016**	**0.007**	**0.0210**	**0.146**	**11.310**	**2.978**	**0.0002**	**0.353**
	Prop	46	**0.018**	**0.007**	**0.0075**	**0.171**	**12.860**	**2.495**	** < 0.0001**	**0.418**
	Cover	46	0.078	0.044	0.0846	0.061	**58.018**	**17.712**	**0.0020**	**0.183**
Non-native allergenics	Richness	54	**0.010**	**0.004**	**0.0078**	**0.166**	**6.687**	**1.530**	** < 0.0001**	**0.400**
	Prop	54	**0.012**	**0.004**	**0.0064**	**0.169**	**8.280**	**1.534**	** < 0.0001**	**0.462**
	Cover	54	**0.189**	**0.052**	**0.0006**	**0.198**	**54.305**	**21.125**	**0.0129**	**0.109**

The proportion, richness and cover of non-native allergenic species increased with urbanisation (Fig. 2c, e), in particular for neophytes (Table 1). The highest neophyte allergenic cover (up to 31.1%) was in grasslands which were both highly neophyte invaded and highly urbanised (positive interaction term: proportion of neophyte *x*% impervious surfaces; cf. Table S4). There was generally a low richness of allergenic neophytes per plots (< 4 species per plot). While *Medicago x varia* was the most frequent allergenic neophyte in these grasslands, the observed increase in allergenic neophyte richness with urbanisation was mostly the result of *Ambrosia coronopifolia*, *Solidago canadensis* and *Plantago arenaria* co-occurring more frequently in the more urban grasslands. Unsurprisingly, richness and cover of allergenic non-natives also increased with the proportion of neophytes (Table 1, Fig. 2d, f). By contrast, the absolute number and cover of native allergenic species did not show any significant trend with urbanisation or plant invasion.

### Potential allergenic value of communities

**Fig. 3:**
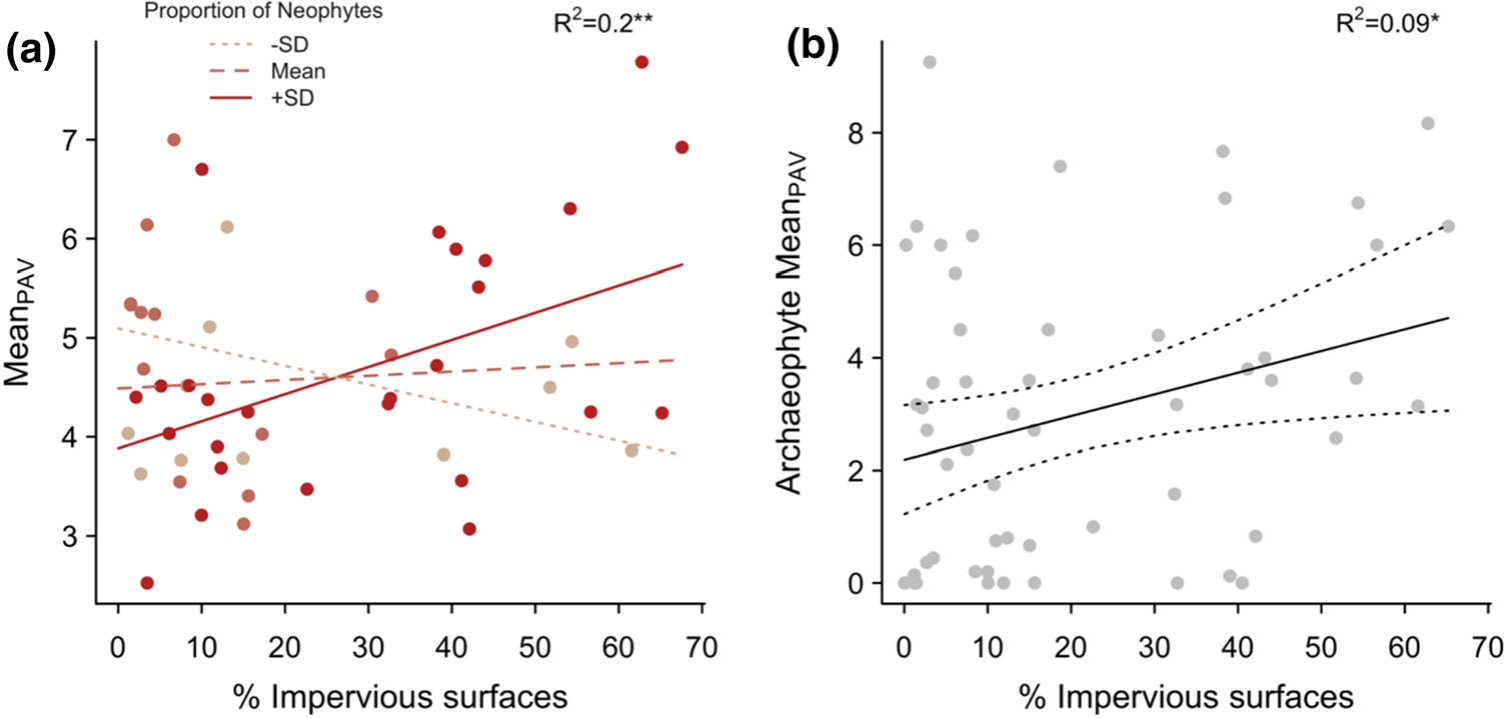
Potential allergenic value (PAV) of grassland communities along the urbanisation gradient (% Impervious surfaces). **a** Mean_PAV_ increased with urbanisation when the proportion of neophytes was high. This positive interaction is illustrated with partial regression lines for three values of proportion of neophytes (*− SD* = 0.016, *Mean* = 0.065, + SD = 0.113). Statistics for the linear model with interaction are indicated. **b** Mean PAV of archaeophytes present in each community also increased with urbanisation. Statistics for the linear model are indicated (details in Table S3). Statistical significance is indicated with stars: *P < 0.05; **P < 0.01

The mean potential allergenic value of grassland communities (Mean_PAV_) ranged from 2.5 to 7.8, while the abundance-weighted means (CWM_PAV_) reached much higher values, up to 22.2 (i.e. 62% of the potential maximum score of 36) for plots dominated by major allergens such as the native grasses *Festuca brevipila* or *Elymus repens,* or the native Asteraceae *Artemisia campestris*. Mean_PAV_ tended to increase when high levels of urbanisation were associated with high levels of neophyte invasion (Fig. 3a; linear model with positive interaction: *df* = 52, *R*^2^ = 0.20, *P* = 0.0092). Considered separately, however, neither the urbanisation nor the invasion gradient was associated with trends in Mean_PAV_ (details in Table S3). When taking species cover into account (CWM_PAV_), the interaction was no longer significant, though overall trends remained similar (Fig. S2). Non-natives, however, and in particular archaeophytes, showed an increase in mean PAV (weighted and unweighted) with urbanisation (Fig. 3b; Table S3). This trend in mean PAV was in part driven by an increase in richness (Poisson regression: Nagelkerke’s *R*^2^ = 0.11, *df* = 54, *P* = 0.0327) and cover (*R*^2^ = 0.07, *df* = 54, *P* = 0.0461) of wind-pollinated non-native allergenics with urbanisation, a trend which was absent for natives.

### Allergen molecule diversity

Total allergen molecule richness of communities was not related to urbanisation (% impervious surfaces) nor to the proportion of neophytes (Table 2). We did, however, detect a significant turnover in the composition of allergen molecules (distance-based redundancy analyses, *r*^2^ = 0.06, *P*  = 0.003) and of allergen families (*r*^2^ = 0.09, *P* = 0.003) along both gradients of urbanisation and proportion of neophytes (cf. details of beta-dissimilarity analyses in Appendix S4).

**Table 2:** Models for allergen molecule and allergen family richness as a function of urbanisation and neophyte invasion. Each GLM was fitted independently using negative binomial distributions for molecule (*Rich.*) and allergen family richness (*Fam.Rich*), or quasi-binomial distributions for proportions of allergenics (*Prop.*). Beta coefficients (and standard errors) are indicated, along with Nagelkerke’s pseudo-*R*^2^ and a *P* value for the likelihood-ratio test against the null model. Statistically significant (*P* < 0.05) models are highlighted in bold

Species	Metric	*n*	% Impervious surfaces				Proportion of Neophytes			
			Coef	SE	*R*^2^	*P*	Coef	SE	*R*^2^	*P*
All	Rich	54	0.002	0.002	0.02	0.3990	1.12	0.76	0.06	0.1487
	Fam.Rich	54	0.002	0.002	0.04	0.1742	1.27	0.66	0.08	0.0562
Natives	Rich	54	− 0.001	0.002	0.00	0.7871	− 0.28	0.82	0.00	0.7382
	Prop	54	− 0.009	0.005	0.35	0.0576	**− 5.88**	**1.84**	**0.67**	**0.0014**
	Fam.Rich	54	0.000	0.002	0.00	0.9107	1.20	0.78	0.08	0.1271
Archaeo	Rich	53	0.007	0.007	0.03	0.2572	4.14	2.40	0.07	0.0953
	Prop	53	0.006	0.005	0.14	0.2397	**3.34**	**1.95**	**0.26**	**0.0874**
	Fam.Rich	53	**0.006**	**0.002**	**0.13**	**0.0060**	**2.00**	**0.89**	**0.09**	**0.0259**
Neophytes	Rich	48	0.017	0.011	0.07	0.1326	**17.26**	**4.63**	**0.26**	**0.003**
	Prop	48	**0.018**	**0.008**	**0.39**	**0.0297**	**11.10**	**3.20**	**0.68**	**0.0006**
	Fam.Rich	46	**0.018**	**0.004**	**0.41**	** < 0.0001**	**8.24**	**1.47**	**0.43**	** < 0.0001**
All non-natives	Rich	54	0.009	0.006	0.06	0.1091	**6.11**	**2.02**	**0.18**	**0.0056**
	Prop	54	0.009	0.005	0.35	0.0576	**5.88**	**1.84**	**0.67**	**0.0014**
	Fam.Rich	54	**0.005**	**0.002**	**0.12**	**0.0071**	**2.84**	**0.81**	**0.19**	**0.0006**

The proportion of neophyte allergens increased both with urbanisation and the proportion of neophytes (Table 2). This increase in neophyte allergens with urbanisation was accompanied by a parallel increase in the number of unique protein families of allergens contributed by neophytes and by archaeophytes. By contrast, for natives, neither allergen richness nor allergen family richness varied along either gradient. Low levels of spatial autocorrelation were found when modelling the number of allergen families of natives (Moran’s *I* = − 0.05, *P* = 0.0030) and archaeophytes (*I* = − 0.04, *P* = 0.0172), but not neophytes (*I* = − 0.01, *P* = 0.3138), along the urbanisation gradient, indicating that the trend found for archaeophyte allergen families needs to be interpreted with some caution.

Unsurprisingly, allergen molecule richness was tightly correlated to the number of allergenic species present in the plot (*ρ* = 0.9, *n* = 56, *P* < 0.0001), and tended to increase with taxonomic family richness, in particular for the group of neophyte allergens (*ρ* = 0.64, *n* = 50, *P* < 0.001; details of correlations in Table S2a). Moreover, allergen protein family richness also increased with taxonomic family richness (Table S2b), indicating that the more diverse these grasslands are taxonomically, the more diverse the biochemical spectrum of allergens produced. When looking more closely at this relationship, however, it only held for the subsets of neophytes (*ρ* = 0.67, *n* = 56, *P* < 0.0001) and archaeophytes (*ρ* = 0.37, *n* = 56, *P* = 0.0056), but not for native species (*ρ* = 0.16, *n* = 56, *P* = 0.2382).

### Timing and length of pollen production in urban grasslands

**Fig. 4:**
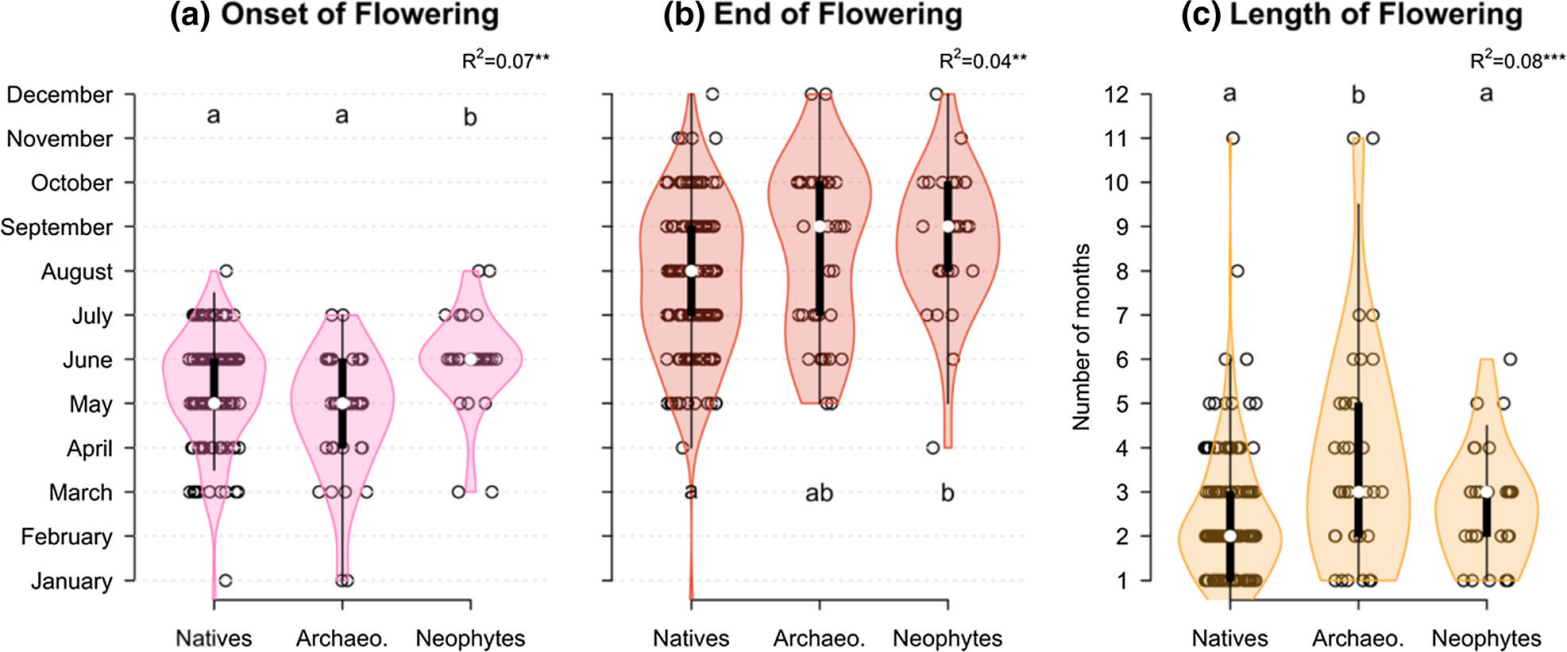
Flowering phenology of 216 urban grassland species divided by introduction status. Panels represent the distribution of **a** the first month of flowering, **b** the last month of flowering and **c** flowering length (in months). Differences between groups (natives, archaeophytes, neophytes) were tested with a bootstrapped non-parametric ANOVA. Post-hoc differences between groups are represented by letters based on bootstrap confidence intervals. Significance levels as indicated previously

Based on the flowering phenology of the 216 grassland species for Germany, allergy season may start as early as March in most plots (or even January in some grasslands when counting the relatively low allergenic and infrequent native *Poa annua*). Natives and archaeophytes generally flowered first, followed on average a month later by neophytes (bootstrapped non-parametric ANOVA; Fig. 4). In addition, both archaeophytes and neophytes flowered until later in the year. As a result, archaeophytes tended to have a longer flowering season (3.6 months, 90% CI 3–4.1) than natives (2.4 months, 2.3–2.7) or neophytes (2.7 months, 2.4–3).

**Fig. 5:**
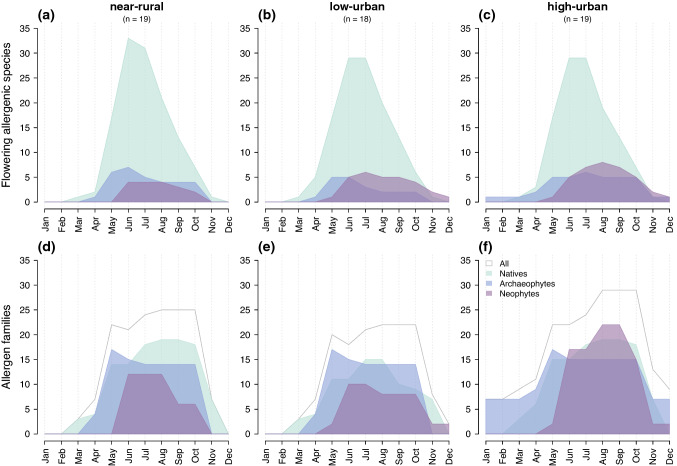
Seasonal distribution of flowering allergenic species and allergen families in Berlin grasslands. The 56 plots of grasslands were clustered in three balanced groups (*n* number of grassland plots per cluster) according to the % of impervious surfaces in a 500 m buffer: < 7% for near rural; between 7 and 30% for low urban; > 30% for high urban. **a**–**c** Potential number of allergenic species flowering each month in grassland communities. For each month, the expected number of unique flowering allergenic species present across a grassland cluster was calculated based on species flowering phenology. Species were divided by introduction status: natives (green), archaeophytes (blue) and neophytes (purple). **d**–**f** Number of unique protein allergen families potentially produced per month, calculated for each floristic status and grassland cluster. Because some protein families were redundant between floristic status groups, the total number of unique allergen families (grey line) is not equal to the sum of the three groups

Allergy season (i.e. the flowering period of allergenic species in a community) tended to end later with increasing urbanisation (Spearman’s *ρ* = 0.32, *n* = 56, *P* = 0.0159) and a higher proportion of neophytes (*ρ* = 0.44, *n* = 56, *P* < 0.0001). The peak of flowering for allergenic neophyte tended to occur significantly later (in August instead of June; Fig. 5c) towards the more urban (*ρ* = 0.39, *n* = 51, *P* = 0.0052) and the more neophyte-invaded plots (*ρ* = 0.28, *n* = 51, *P* = 0.0454). It should be noted that since the phenology data used in this study is based on trait databases at the species level for the whole of Germany and not on actual phenology of populations in Berlin, any trend detected only reflects changes in species composition.

Taking into account flowering phenology, we calculated the mean monthly number of allergenic species flowering simultaneously in each plot (proportional to the area under the curve represented in Fig. 5a–c). The results (details of models in Table S6) are consistent with the overall community patterns previously obtained when ignoring phenology. Mean monthly allergenic per cent cover increased with urbanisation (*df* = 54, *R*^2^ = 0.11, *P* = 0.0117), again a pattern mostly driven by non-natives (*df* = 54, *R*^2^ = 0.21, *P* < 0.0001; Fig. 5a–c). We found a parallel increase with urbanisation for the mean monthly number of allergen molecules produced by non-natives (*df* = 54, *R*^2^ = 0.13, *P* = 0.0054) as well as the mean monthly number of allergen families produced (*df* = 54, *R*^2^ = 0.09, *P* = 0.0213; illustrated in Fig. 5d–f). Native allergenic species showed none of these trends (cf. Table S6).

## Discussion

Our study shows that (a) as urbanisation increases, dry grasslands in Berlin become more allergenic, both in terms of allergen diversity and cover; and (b) this increase is in large part driven by non-native plants. To our knowledge, this study is the first to provide a quantification of allergenicity at the plant community level for a standardised model ecosystem (i.e. dry grassland) along an urbanisation gradient. By combining community vegetation surveys with available data on species allergenic and phenological traits, we were able to compare grassland communities in terms of richness, abundance and turnover of allergenic species and their allergens. We further showed that the spectrum of allergen molecules expanded with increasing neophyte invasion, as neophyte species tended to introduce a higher diversity of biochemical families of allergens than the native urban flora. Since neophytes often flower later in the season, they also extended the allergenic season of the more urban and invaded grasslands later into autumn.

The last few years have seen a surge of interest in quantifying allergenicity of urban green spaces (Hruska 2003; Cariñanos et al. 2014, 2017; Kasprzyk et al. 2019; Velasco-Jiménez et al. 2020; Aerts et al. 2021). These studies, like our own, are limited by a lack of standardised allergenicity scales, uneven quality of evidence across allergen datasets and knowledge gaps (certain species being more studied than others), which altogether significantly affect risk assessments (Sousa-Silva et al. 2021; Suanno et al. 2021). To address these issues, we adopted a systematic data collection approach based on evidence from molecular allergen databases and published immunological reports rather than expert knowledge or previously published lists of allergenic species. Systematic extrapolations based phylogenetic conservatism allowed us to fill in some of the data gaps, providing a conservative estimation of both allergenicity and biochemical diversity. This reproducible workflow, for which we provide the full code online (cf. methods), can be readily replicated and updated with other urban vegetation datasets, providing a common basis for comparison across cities and regions.

### Novel urban grasslands are more allergenic

Towards the more urbanised grasslands of Berlin, allergenic species cover increased by almost 20%, and there was on average a higher cover of allergenic plants flowering at any given time. Moreover, where urbanisation was coupled with high neophyte invasions, grassland species had on average a higher potential allergenic value (PAV). Our results, thus, show for the first time that urbanisation and biological invasions can be associated to potential hotspots of allergenic pollen production in grassland ecosystems.

Several recent studies have documented a high potential allergenic risk for urban parks and gardens (Cariñanos et al. 2017; Kasprzyk et al. 2019; Velasco-Jiménez et al. 2020), but none to our knowledge has yet studied a standardised ecosystem, nor quantified the variation in risk along an urbanisation gradient. In our lightly managed study system, the observed gradual turnover in allergen species composition reflects a shift in the underlying mechanisms driving plant community assembly in these grassland ecosystems. Our finding that local factors—amount of built-up areas around the grassland patch and level of neophyte invasion—were significant predictors of allergenicity supports previous observations that urban pollen environments are determined by local habitat filters (Rodríguez-Rajo et al. 2010; Katz and Carey 2014; Werchan et al. 2017).

While natives constitute the vast majority (c. 80%) of allergenic species in these dry grasslands, increase in allergenic cover with urbanisation was driven by the gradual increase in cover and richness of non-native allergenics. A similar increase in the frequency of non-native allergenics with urbanisation has been noted in a few studies: in Beijing (China), residential areas had a higher proportion of non-native allergenics than urban parks and forests (Mao et al. 2013); and in Córdoba (Spain), ornamental exotic pollen appeared more abundant in both urban centres and residential areas (Cariñanos et al. 2002). Other allergenic weeds such as ragweed (*Ambrosia* spp.) increase in abundance in urban areas (Ziska et al. 2003) both in their native and non-native range (Katz and Carey 2014).

Not only did non-native allergenics become more abundant, but their average potential allergenic value (PAV) also increased with urbanisation, especially within the group of archaeophytes. This reflected an increase in frequency of non-native species with traits associated to higher PAV: longer flowering seasons (for archaeophytes) and wind pollination. Wolf et al. (2020) found a similar increase in wind-pollinated neophytes across German cities and suggest it may be the result of either a decrease in pollinators or a secondary effect of the success of a few typically wind-dispersed plant families (i.e. Poaceae and Chenopodiaceae) in urban areas.

Nevertheless, half of non-natives classified as allergenics in Berlin grasslands were insect pollinated, thus, scoring low PAVs, and non-natives were not on average more frequently or more severely allergenic than natives. Thus, high levels of plant invasions in themselves do not predict high allergenicity, and merely counting the number of allergenic species does not directly relate to potential allergenic value. Quantifying the PAV of species offers a useful proxy for assessing allergenic potential, though it remains imprecise and limited by data quality. In particular, it does not reflect the variation in pollen size and number within classes of pollination syndromes, which influence the spatial distribution of pollen loads (Werchan et al. 2017), nor the within-species variation in the timing, quantity and allergenicity of pollen production which are affected by local factors such as a warmer microclimate, rising CO_2_ levels and pollution, all intrinsically tied to urbanisation (Ziska et al. 2003; Barnes 2018).

Our study likely presents a rather conservative picture of the potential allergenicity of herbaceous urban green spaces. Berlin dry grasslands are characterised by low to moderate disturbances and showed a relatively low proportion of neophytes (< 20%), only weakly correlated with urbanisation. Neophytes reach higher proportions in more disturbed types of urban vegetation in Berlin (e.g. up to 32% neophytes in ruderal vegetation; Kowarik 1990) and may sometimes reach up to 50% in other cities (Pyšek 1998). Given our results, we would predict an even steeper increase in allergenicity when looking at those more invaded herbaceous urban ecosystems.

### A broader spectrum of allergen molecules in urban grasslands

Botanical taxonomic diversity of airborne pollens is typically monitored as a proxy for the spectrum of allergen molecules that humans are exposed to (Brennan et al. 2019). However, taxonomy is an imperfect predictor of allergen molecular differences and cross-reactivities, and classification of allergens into protein families might provide a more accurate picture (Radauer and Breiteneder 2006). In our study, trends in allergen protein family richness were not predictable from grassland species richness: high numbers of allergenic species could still mean low numbers of allergen families if plants all produced similar allergens.

Across Berlin dry grasslands, we found an increase in non-native allergen family richness with urbanisation. By contrast, native allergens showed no such trend along invasion or urbanisation gradients. In general, the pool of neophytes produced a more biochemically diverse range of allergens than the pool of natives or archaeophytes: neophytes contributed on average twice the number of unique protein families than natives. This suggests that neophyte pollens tend to be more biochemically distinct from each other. Such a difference between natives and non-natives cannot be easily explained by simply looking at taxonomic families. Natives did not show a lower taxonomic family richness than non-natives: they were in fact much more diverse in terms of species and family numbers. Neophytes also did not belong disproportionately to those taxonomic plant families with more unique and diverse allergen molecules, such as Asteraceae (Radauer and Breiteneder 2006; Tong and Lin 2015).

A possible hypothesis for the high biochemical diversity of neophyte allergens may be the variety of species’ biogeographical and evolutionary origins. Allergen molecule distribution across plants appears in large part explained by recent evolutionary events of gene duplication and selection (Chen et al. 2016). One might, thus, expect biochemical differences to have accumulated recently between evolutionary lineages *within taxonomic families* separated by continents, either via genetic drift or different selection pressure, creating evolutionary legacies (sensu Cavender-Bares et al. 2016) at a fine phylogenetic scale. This is supported by the fact that only for non-natives did taxonomic family richness correlate with protein family richness. Further exploring this hypothesis would require a precisely dated phylogeny resolved at the species level, allowing to test whether phylogenetic diversity is a good predictor of allergen biochemical diversity in communities.

### Extended allergy season in the urban core

Allergy season extended later into autumn in the more urban grasslands, as late blooming allergenic species became more frequent. This shift in phenology was expected due to the warmer microclimate created by the urban heat-island effect, which extends the favourable growing period (Kowarik et al. 2011; Jochner and Menzel 2015) and, thus, opens new phenological niches for late bloomers. Late-flowering allergenic species in Berlin grasslands belonged mainly to the Asteraceae family (Fig. S3) and comprised severe allergenic species, such as *Artemisia* spp. and *Ambrosia* spp., as well as more moderate ones, such as *Solidago canadensis* or *Tanacetum vulgare*. Allergenic *Artemisia* invaders from warmer regions have been documented in other European cities (e.g. Cristofori et al. 2020), suggesting that this genus may be of general concern in these regions, in particular as climate change will accentuate urban warming and favour warm-loving late bloomers (Li et al. 2020).

In our study, the phenological shift of urban communities towards later flowering was in large part driven by the increase in non-native species, with neophytes flowering later and archaeophytes flowering longer. Theory in invasion biology predicts higher establishment success for species able to occupy an empty temporal niche in the resident community (Wolkovich and Cleland 2014), a hypothesis supported empirically in several grassland systems (e.g. Godoy et al. 2009; Fried et al. 2019). Our results also support this hypothesis and provide a general prediction that late blooming non-natives may be particularly successful urban invaders in temperate regions. This possible role of phenological differences in promoting invasion success suggests that the timing and intensity of management in urban grasslands may play a critical role in driving future abundances of non-native urban allergens.

Phenological trends in our study ignored potential intraspecific differences in species phenology between urban and rural populations. Warmer temperatures in the urban core shift the flowering phenology of species towards earlier flowering (Jochner and Menzel 2015), but also longer flowering periods involving multiple flowering events later into autumn (Li et al. 2020), thereby extending the allergy season (Rodríguez-Rajo et al. 2010; Katz et al. 2019). The phenological differences observed in our study are, therefore, likely to be relatively conservative, and even longer allergy seasons may be expected in the more urbanised areas.

### Perspectives for urban nature management

The benefits of urban biodiversity need to be balanced with possible disservices to human populations, such as allergies (Potgieter et al. 2017). While the regional amount of airborne pollen in city centres may decrease as vegetation is replaced by built-up areas (Rodríguez-Rajo et al. 2010; Bosch-Cano et al. 2011; Katz et al. 2019), local hotspots such as the novel urban grasslands in our study may have a large impact on allergy sufferers (Cariñanos et al. 2002), especially in cities where the population is more susceptible to allergies than in rural areas (D’Amato et al. 2007; Ziska and Beggs 2012; von Mutius 2016).

Major allergenic tree species in city parks and streets may be actively removed (Aerts et al. 2021) or excluded when designing new urban green spaces (Cariñanos and Casares-Porcel 2011), but it is an altogether different challenge to control the spread of self-seeded herbaceous species. For recently introduced species, such as ragweed in Europe, containment actions may still have some measure of success, in particular when overlapping goals with biological invasion control provide a legal and financial framework (Buters et al. 2015). But our study highlights the need to consider not only invasive neophytes but also the well-established resident allergenic flora of natives and archaeophytes in cities. For instance, *Artemisia* species pose a high allergy risk and are increasingly abundant in many European cities (Bogawski et al. 2016; Cristofori et al. 2020), but, being already widespread in the urban flora, are not candidates for removal actions.

A second approach to mitigating urban allergens is at the habitat scale, adopting practices favouring low-allergenicity ecosystems. In our study, grasslands with the highest potential allergenic value were both very urban and highly invaded by neophytes. In these grasslands, neophytes were not themselves more allergenic, but rather acted as indicators of habitat conditions—i.e. an increase in man-made disturbances (Kowarik 1990)—fostering novel plant communities rich in allergenic species, both native and non-native. Reducing disturbances and encouraging succession from pioneer ruderal vegetation or, alternatively, intensively managed lawns, to diverse semi-natural meadows within the city may decrease the local abundance and impact of major allergens (Katz and Carey 2014; Watson et al. 2020). Previous studies have mentioned frequent mowing as a strategy to avoid flowering of herbaceous species (e.g. Cariñanos et al. 2017), but this does not take into consideration some species’ biology (e.g. ragweed may still resprout and flower after mowing; Essl et al. 2015) nor the community dynamics of these systems. In fact, reducing mowing frequency is known to help maintain a diverse herbaceous layer, while allowing successional processes to replace ruderal weedy neophytes with a diversity of more competitive long-term natives (Chollet et al. 2018; Norton et al. 2019). Controlled sowing, perhaps associated with soil improvement (e.g. via mycorrhiza inoculation), have also helped convert lawns into meadows increasing native biodiversity (Fischer et al. 2013a; Norton et al. 2019), though care must be taken when sowing seeds: standard pollinator flower mixes often involve a high proportion of neophytes, which may not only be potentially allergenic, but also change the overall dynamic of grassland communities (Johnson et al. 2017).

However, overall increase in allergen abundance and diversity with urbanisation may not have only deleterious effects. Predicting the long-term effect of allergen exposure on human populations is challenging (Linneberg 2008; Bergmann et al. 2012; Haahtela et al. 2021), and increased prevalence of asthma and allergies in cities and westernised societies has been related to *reduced* pollen exposure, to the point where some researchers question whether we are, in fact, getting enough allergens in our environment (Linneberg 2008). There is mounting evidence for the *biodiversity hypothesis* (Hanski et al. 2012), stating that reduced exposure to a diversity of microbes, which is enhanced by broader biodiversity, may reduce our immune tolerance. Several recent studies in different cities around the world support the protective effect of growing up and living in greener areas of the city (Dzhambov et al. 2021; Paciência et al. 2021). However, some studies have also found that growing up in areas of a city with a high number of allergenic trees may increase allergy prevalence later in life (Markevych et al. 2020). This apparent contradiction may be a case where the effect of the abundance of a few high-impact allergens needs to be dissociated from that of overall exposure to allergen diversity. A large-scale study on allergenic rhinitis and asthma in New Zealand found a beneficial effect of a diverse and green environment during childhood (Donovan et al. 2018), but this positive effect became negative when the surrounding vegetation was dominated by low-diversity, non-native-invaded land types. In this light, the increasing allergen diversity detected in Berlin urban grasslands may be less a cause for concern than the overall decrease in biodiverse green areas in the city centre.

Finally, other urban factors exacerbating the allergy problem in cities such as air pollution and urban warming may be more important in reducing allergy cases than eradicating allergenic species (Reinmuth-Selzle et al. 2017), and these can be mitigated by increasing the surface and complexity of green areas (Sandifer et al. 2015). As the global population becomes more urban, urban green areas will become the predominant type of nature to which children are exposed to on a regular basis. The rising concern of urban allergies may only be addressed by tackling multiple factors, from reducing pollution to managing urban nature. Our study shows that urban grasslands, one of the most diverse urban novel ecosystems, can create hotspots of allergenic pollen when local percentages of built-up areas and man-made disturbances are high. While reducing the quantity of severe allergenic pollens in cities remains a desirable goal, it may be better addressed by mitigating those local factors creating allergenicity hotspots, while providing an overall greener and more diverse environment for populations growing up in cities.

## Supplementary Information

Below is the link to the electronic supplementary material.

Supplementary file1 (PDF 916 KB)
