# Supplementary material for: Grassland allergenicity increases with urbanisation and plant invasions
Source: Ambio. 2022 May 20;51(11):2261–77. doi: 10.1007/s13280-022-01741-z (PMC9481851; doi:10.1007/s13280-022-01741-z)
Supplement: Supplementary file 1 — Supplementary file1 (PDF 916 KB) [file 13280_2022_1741_MOESM1_ESM.pdf]

Ambio

Electronic Supplementary Material

This supplementary material has not been peer-reviewed.

Title: Grassland allergenicity increases with urbanisation and plant invasions

Maud Bernard-Verdier, Birgit Seitz, Sascha Buchholz, Ingo Kowarik, Sara Lasunción Mejía and Jonathan M. Jeschke

Appendix S1: Supplementary Tables 2
Appendix S2: Supplementary Figures 9
Appendix S3: Collecting allergenicity data 12
Appendix S4: Turnover in allergen composition 17

Appendix S1: Supplementary Tables

Table S1. Spearman rank correlations between components of species richness (SR) and allergenic species richness (SR_{Allerg}). ____ 3

Table S2. Spearman rank correlations between components of species richness (SR) and allergen molecule richness (AR). ____ 3

Table S3. Trends in community mean Potential Allergenic Value (PAV), both unweighted ($Mean_{PAV}$) and weighted by abundances (CWM_{PAV}).. ____ 4

Table S4. Best models explaining allergenic species richness, proportion and cumulative cover as a function of urbanisation and biotic novelty across the 56 Berlin grasslands. ____ 5

Table S5. Best models explaining allergen molecule richness, allergen proportion and allergen family richness as a function of urbanisation and proportion of neophytes. ____ 6

Table S6. Mean monthly allergen richness and cover as a function of urbanization and biotic novelty. ____ 7

Table S7. List of the ten most allergenic species per introduction status category in Berlin dry grasslands. ____ 8

Table S1. Spearman rank correlations between components of species richness (SR) and allergenic species richness (SR_{Allerg}). Spearman's rho is provided, with significant levels indicated by stars (***, P < 0.001; **, P < 0.01; *, P < 0.05; ns, P ≥ 0.05). All correlations include 56 plots.

		Species richness				*Allergenic species richness*		
		**SR**	**SR.nat**	**SR.arch**	**SR.neo**	**SR_{Allerg}**	**Neo. SR_{Allerg}**	**Arch. SR_{Allerg}**
Species richness	**SR.nat**	0.92 ***						
	**SR.arch**	0.66 ***	0.43 ***					
	**SR.neo**	0.39 **	0.16 ns	0.31 *				
Allergenic species richness	**SR_{Allerg}**	0.84 ***	0.77 ***	0.53 ***	0.43 ***			
	**Neo. SR_{Allerg}**	0.23 ns	0.07 ns	0.22 ns	0.69 ***	0.39 **		
	**Arch. SR_{Allerg}**	0.34 **	0.17 ns	0.5 ***	0.37 **	0.41 **	0.14 ns	
	**Nat. SR_{Allerg}**	0.77 ***	0.84 ***	0.36 **	0.14 ns	0.84 ***	0.11 ns	0.01 ns

Table S2a. Spearman rank correlations between components of allergen molecule richness (AR), species richness (SR), and taxonomic family richness (FR). Correlations with taxonomic family richness (FR) were calculated for each subset of native, archaeophyte or neophyte species. Spearman's *rho* is provided, with stars indicating significance levels (***, P < 0.001; **, P < 0.01; *, P < 0.05; ns, P ≥ 0.05). For neophyte values, correlations are calculated on 50 plots only, excluding 6 plots which had no neophytes.

		Species richness				*Family richness*	*Allergen molecule richness*		
		**SR**	**SR.nat**	**SR.arch**	**SR.neo**	**FR**	**AR**	**AR.neo**	**AR.arch**
Allergen molecule richness	**AR**	0.8 ***	0.71 ***	0.54 ***	0.39 **	0.44 ***			
	**AR.neo**	0.14 ns	-0.03 ns	0.18 ns	0.62 ***	0.64 ***	0.29 *		
	**AR.arch**	0.41 **	0.25 ns	0.51 ***	0.34 *	0.4 **	0.54 ***	0.15 ns	
	**AR.nat**	0.72 ***	0.72 ***	0.37 **	0.16 ns	0.34 *	0.82 ***	-0.01 ns	0.06 ns

Table S3b. Spearman rank correlations between components of allergen biochemical family richness (AFR), species richness (SR), taxonomic family richness (FR) and Rao's quadratic entropy (Rao). Correlations with taxonomic family richness (FR) were calculated for each subset of native, archaeophyte or neophyte species. Spearman's *rho* is provided, with stars indicating significance levels (***, P < 0.001; **, P < 0.01; *, P < 0.05; ns, P ≥ 0.05). For neophyte values, correlations are calculated on 50 plots only, excluding 6 plots which had no neophytes.

		Species richness				*Family richness*	*Allergen Family richness*		
		**SR**	**SR.nat**	**SR.arch**	**SR.neo**	**FR**	**AFR**	**AFR.neo**	**AFR.arch**
Allergen family richness	**AFR**	0.56 ***	0.5 ***	0.38 **	0.26 ns	0.36 **			
	**AFR.neo**	0.13 ns	-0.03 ns	0.17 ns	0.61 ***	0.63 ***	0.3 *		
	**AFR.arch**	0.5 ***	0.4 **	0.47 ***	0.27 *	0.37 **	0.64 ***	0.22 ns	
	**AFR.nat**	0.39 **	0.39 **	0.12 ns	0.18 ns	0.16 ns	0.5 ***	-0.13 ns	0.25 ns

Table S4. Trends in community mean Potential Allergenic Value (PAV), both unweighted ($Mean_{PAV}$) and weighted by abundances (CWM_{PAV}). Two predictors were tested independently: the percentage of impervious surfaces in a 500m buffer and the proportion of neophytes in the grassland community. Models were fitted for different subsets of species (All, Natives, Archaeophytes, Neophytes, and all non-natives (Archaeophytes + Neophytes). Significant models (P < 0.05) are in bold.

				% Impervious surfaces				Proportion of Neophytes			
**Subset of species**	**metric**	**mean (± sd)**	**df**[†]	*coef*	*se*	*P*	R^2	*coef*	*se*	*P*	R^2
All	mean.pav	4.77 (± 1.2)	54	0.008	0.008	0.3427	0.017	0.29	3.08	0.93	0.000
	cwm.pav	5.61 (± 3.83)	54	-0.012	0.025	0.6342	0.004	-5.54	9.80	0.5740	0.006
Natives	mean.pav	5.42 (± 1.46)	54	0.003	0.010	0.7746	0.002	4.00	3.72	0.2861	0.021
	cwm.pav	9.38 (± 3.19)	54	0.012	0.021	0.5794	0.006	4.65	8.16	0.5716	0.006
Archaeophytes	mean.pav	2.99 (± 2.61)	53	**0.039**	**0.017**	**0.0300**	**0.086**	3.83	6.79	0.5749	0.006
	cwm.pav	4.2 (± 4.67)	53	**0.094**	**0.030**	**0.0028**	**0.157**	6.95	12.16	0.5702	0.006
Neophytes	mean.pav	3.7 (± 6.02)	48	0.001	0.042	0.9793	0.000	-4.06	18.17	0.8239	0.001
	cwm.pav	4.13 (± 6.88)	48	-0.001	0.048	0.9908	0.000	-2.00	20.78	0.9237	0.000
All non-natives	mean.pav	3.1 (± 2.24)	54	0.025	0.014	0.0873	0.053	2.10	5.75	0.7156	0.002
	cwm.pav	3.57 (± 3.95)	54	**0.054**	**0.025**	**0.0367**	**0.078**	-2.19	10.12	0.8292	0.001

[†] Residual degrees of freedom

Table S5. Best models explaining allergenic species richness, proportion and cumulative cover as a function of urbanisation and biotic novelty across the 56 Berlin grasslands. Statistics from linear (cover), and Binomial (proportions) or Poisson (richness) models are presented. Best models based on AICc were selected from a full model with interactions between the % of impervious surfaces (*Imperv.*) and the proportion of neophytes (prop.neo). R^2 values for GLMs are Nagelkerke pseudo-R^2, and P-values correspond to a Likelihood-ratio test compared to a null model. Partial R^2 calculated with Wald statistics are presented for each predictor in the best models. Non-native allergenics correspond to the sum of Neophyte and Archaeophyte allergenics**.**

						**Predictors**			
**Subset of species**	**metric**	**best model**	**df**[†]	**R^2**	**P**	*variable*	*coef*	*SE*	*pR^2*
**Allergenic species**	*richness*	*null*	56						
	cover	Imperv.	56	0.07	0.0483	Imperv.	0.26	0.13	0.07
**Native allergenics**	*richness*	*null*	56						
	proportion	prop.neo	56	0.46	0.0000	prop.neo	-8.28	1.84	0.35
	cover	*null*	56						
**Archaeophyte allergenics**	*richness*	prop.neo	55	0.15	0.0149	prop.neo	4.55	1.85	0.13
	proportion	prop.neo	55	0.13	0.0294	Imperv.	0.12	0.04	0.15
	cover	Imperv.	55	0.15	0.0031	prop.neo	4.55	2.08	0.13
**Neophyte allergenics**	*richness*	prop.neo	50	0.35	0.0002	prop.neo	11.31	2.98	0.37
	proportion	prop.neo	48	0.42	0.0000	prop.neo	12.86	3.14	0.34
	cover	prop.neo * Imperv.	50	0.31	0.0005	prop.neo	-9.28	28.13	0.00
						Imperv.	-0.16	0.08	0.08
						prop.neo:Imperv.	2.10	0.75	0.15
**Non-native allergenics**	*richness*	prop.neo	56	0.40	0.0000	prop.neo	6.69	1.53	0.35
	proportion	prop.neo	56	0.46	0.0000	prop.neo	8.28	1.84	0.35
	cover	Imperv.	56	0.20	0.0006	Imperv.	0.19	0.05	0.20

[†] Residual degrees of freedom

Table S6. Best models explaining allergen molecule richness, allergen proportion and allergen family richness as a function of urbanisation and proportion of neophytes. Model selection according to AICc was carried out on an exhaustive set of Negative Binomial models (Richness and Family Richness) or Quasibinomial models (Proportion) with interactions, starting from the full model: (metric ~ % impervious surfaces * proportion of neophytes). When the top models (ΔAICc < 2) did not include the null model, then the best model was the one with lowest AICc. Nagelkerke's pseudo-R2 and the P-value for a likelihood ratio test against the null model are indicated for each best model. Coefficients, standard errors and partial R^2 of the selected predictors are also provided.

						**Predictors**			
**Subset of species**	**metric**	**Best model**	**df**†	**P**	**R2**	*variable*	*coef*	*SE*	*pR2*
All	*Rich.*	*null*	55						
	Fam.Rich	*null*	55						
Natives	*Rich.*	*null*	55						
	Prop.	Imperv. * prop.neo	52	<0.0001	0.73	Imperv.	-0.01	0.00	0.07
						prop.neo	-8.16	1.28	0.19
						Imperv.:prop.neo	0.10	0.03	0.06
	Fam.Rich	*null*	55						
Archaeophytes	*Rich.*	*null*	54						
	Prop.	Imperv. * prop.neo	51	<0.0001	0.36	Imperv.	0.01	0.00	0.05
						prop.neo	5.37	1.37	0.10
						Imperv.:prop.neo	-0.09	0.04	0.04
	Fam.Rich	Imperv.	53	<0.0001	0.13	Imperv.	0.01	0.00	0.04
Neophytes	*Rich.*	prop.neo	48	0.0030	0.26	prop.neo	17.26	4.63	0.27
	Prop.	Imperv. + prop.neo	47	<0.0001	0.70	Imperv.	0.01	0.00	0.00
						prop.neo	9.60	1.65	0.20
	Fam.Rich	Imperv. + prop.neo	47	<0.0001	0.65	Imperv.	0.01	0.00	0.00
						prop.neo	9.13	1.66	0.22
All Non-Natives	*Rich.*	prop.neo	54	0.0056	0.18	prop.neo	6.11	2.02	0.19
	Prop.	Imperv. * prop.neo	52	<0.0001	0.73	Imperv.	0.01	0.00	0.07
						prop.neo	8.16	1.28	0.19
						Imperv.:prop.neo	-0.10	0.03	0.06
	Fam.Rich	Imperv. * prop.neo	52	0.0001	0.32	Imperv.	0.011	0	0.1
						prop.neo	4.565	1.2	0.1
						Imperv.:prop.neo	-0.09	0	0.1

† Residual degrees of freedom

Table S7. Mean monthly allergen richness and cover as a function of urbanization and biotic novelty. Statistics from linear models are presented for four variables: mean monthly allergenic species richness, species cover, molecule richness and allergen family richness. Best models based on AICc were selected from a full model with interactions between the % of impervious surfaces (*Imperv.*) and the proportion of neophytes (prop.neo). Significant interaction terms in the "best models" were all positive. Independent models fitted for each of two predictors are also presented. P-values correspond to a Likelihood-ratio test compared to the null model. Non-native allergenics correspond to the sum of neophyte and archaeophyte allergenics**.**

		BEST MODELS :				INDEPENDENT MODELS:							
						**% Impervious surfaces**				**Proportion of Neophytes**			
**Species**	**monthly metric**	**n**	**predictors**	**P**	**R2**	*coef*	*SE*	*P*	R^2	*coef*	*SE*	*P*	R^2
All	*Species richness*	56	*null*			0.013	0.008	0.1086	0.05	2.52	3.06	0.4128	0.01
	Species cover	56	Imperv.	0.0117	0.11	**0.012**	**0.005**	**0.0117**	**0.11**	1.50	1.94	0.4426	0.01
	Molecule richness	56	*null*			0.084	0.044	0.0627	0.06	26.62	17.26	0.1288	0.04
	All.Fam richness	56	*null*			0.003	0.001	0.0555	0.07	0.75	0.56	0.1916	0.03
Natives	*Species richness*	56	*null*			-0.001	0.006	0.8785	0.00	-3.09	2.46	0.2147	0.03
	Species cover	56	prop.neo * Imperv.	0.0418	0.14	0.003	0.004	0.4576	0.01	-0.96	1.65	0.5659	0.01
	Molecule richness	56	*null*			0.004	0.029	0.8982	0.00	-1.57	11.21	0.8891	0.00
	All.Fam richness	56	*null*			0.002	0.010	0.8788	0.00	3.50	3.79	0.3597	0.02
Archaeophytes	*Species richness*	55	Imperv.	0.0038	0.15	**0.010**	**0.003**	**0.0038**	**0.15**	**2.78**	**1.25**	**0.0308**	**0.09**
	Species cover	55	Imperv.	0.0020	0.17	**0.017**	**0.005**	**0.002**	**0.17**	1.58	2.11	0.4566	0.01
	Molecule richness	55	Imperv.	0.0057	0.14	**0.074**	**0.026**	**0.0057**	**0.14**	18.87	10.01	0.0649	0.06
	All.Fam richness	55	Imperv.	0.0122	0.11	**0.050**	**0.019**	**0.0122**	**0.11**	8.52	7.58	0.2661	0.02
Neophytes	*Species richness*	50	Imperv. + prop.neo	0.0002	0.31	**0.005**	**0.002**	**0.0102**	**0.13**	**3.23**	**0.77**	**0.0001**	**0.27**
	Species cover	50	prop.neo	0.0039	0.16	0.009	0.006	0.1096	0.05	**7.10**	**2.34**	**0.0039**	**0.16**
	Molecule richness	50	prop.neo	0.0016	0.19	**0.019**	**0.009**	**0.0358**	**0.09**	**12.27**	**3.66**	**0.0016**	**0.19**
	All.Fam richness	50	prop.neo	0.0017	0.19	**0.018**	**0.008**	**0.032**	**0.09**	**11.24**	**3.38**	**0.0017**	**0.19**
All non-natives	*Species richness*	56	Imperv. + prop.neo	0.0000	0.32	**0.014**	**0.004**	**0.0004**	**0.21**	**5.61**	**1.37**	**0.0001**	**0.24**
	Species cover	56	Imperv.	0.0008	0.19	**0.020**	**0.006**	**0.0008**	**0.19**	**5.93**	**2.33**	**0.0137**	**0.11**
	Molecule richness	56	Imperv. + prop.neo	0.0062	0.17	**0.080**	**0.028**	**0.0054**	**0.13**	**28.19**	**10.85**	**0.0121**	**0.11**
	All.Fam richness	56	Imperv.	0.0213	0.09	**0.044**	**0.019**	**0.0213**	**0.09**	12.00	7.33	0.1077	0.05

Table S8. List of the ten most allergenic species per introduction status category in Berlin dry grasslands. Species were ranked by potential allergenic value (PAV) and organised by introduction status (N, neophyte; A, archaeophyte; I, indigenous, aka native). Species frequency of occurrence (Freq.) is measured in number of plots and divided into three subsets of plots along the urbanisation gradient (19 near-rural, 18 low-urban, 19 high-urban, cf. Figure 5 in main text).

Species name[a]	Family	Status[b]	Freq.	Flower.	Poll.	Allerg. Score	PAV
Arrhenatherum elatius	Poaceae	N (1787)	3_2_4	Jun-Oct	wind	3	27
Plantago arenaria	Plantaginaceae	N (1787)	0_0_2	Jun-Sep	wind	3	27
Ambrosia coronopifolia	Asteraceae	N (1946)	0_0_2	Aug-Oct	wind	4	24
Lolium multiflorum	Poaceae	N (1859)	1_0_0	Jun-Aug	wind	3	18
Bromus inermis	Poaceae	N (1787)	2_1_3	Jun-Jul	wind	3	9
Eragrostis minor	Poaceae	N (1953)	0_0_1	Jul-Aug	wind	2	6
Senecio inaequidens	Asteraceae	N (1993)	0_1_1	Jul-Dec	insects	2	6
Senecio vernalis	Asteraceae	N (1859)	0_1_1	May-Nov	insects	2	6
Medicago x varia	Fabaceae	N (1859)	1_1_11	Jun-Sep	insects	1	3
Solidago canadensis	Asteraceae	N (1862)	2_2_1	Aug-Oct	insects	1	2
Plantago lanceolata	Plantaginaceae	A	8_8_12	May-Oct	wind	3	27
Plantago major	Plantaginaceae	A	2_0_0	Jun-Oct	wind	3	27
Digitaria ischaemum	Poaceae	A	1_0_2	Jul-Oct	wind	2	18
Setaria viridis	Poaceae	A	5_2_6	Jun-Oct	wind	2	18
Poa annua	Poaceae	A	0_0_2	Jan-Dec	selfing	3	9
Poa bulbosa	Poaceae	A	1_0_1	May-Jun	wind	3	9
Digitaria sanguinalis	Poaceae	A	0_0_3	Jul-Oct	selfing	2	6
Echium vulgare	Boraginaceae	A	3_2_7	May-Jul	insects	2	4
Arabidopsis thaliana	Brassicaceae	A	12_8_8	Apr-May	selfing	1	1
Artemisia vulgaris	Asteraceae	I	1_3_2	Jul-Nov	wind	4	36
Lolium perenne	Poaceae	I	2_0_4	May-Oct	wind	4	36
Festuca ovina	Poaceae	I	1_1_2	May-Aug	wind	3	27
Artemisia campestris	Asteraceae	I	8_10_5	Aug-Oct	wind	4	24
Dactylis glomerata	Poaceae	I	4_5_7	May-Jul	wind	4	24
Holcus lanatus	Poaceae	I	1_4_0	Jun-Aug	wind	4	24
Chenopodium album	Chenopodiaceae	I	0_0_1	Jul-Oct	wind	2	18
Deschampsia flexuosa	Poaceae	I	2_0_0	Jun-Aug	wind	3	18
Elymus repens	Poaceae	I	13_11_8	Jun-Aug	wind	3	18
Festuca brevipila	Poaceae	I	11_11_17	May-Jul	wind	3	18

[a] accepted name in the *Tropicos* database. [b] with year of introduction in Berlin for neophytes (Seitz *et al.* 2012)

References

Seitz, B., M. Ristow, R. Prasse, B. Machatzi, G. Klemm, R. Böcker, and H. Sukopp. 2012. Der Berliner Florenatlas. *Botanischer Verein von Berlin und Brandenburg Beiheft* 7, 1-533.

Appendix S2: Supplementary Figures

Figure S1. Distribution of allergen molecules and allergen protein families across species. 10

Figure S2. Abundance-weighted potential allergenic value (CWM_{PAV}) of grassland communities along the urbanisation gradient (% Impervious surfaces). 11

Figure S3. Flowering distribution of allergenic species grouped by taxonomic families.. 11

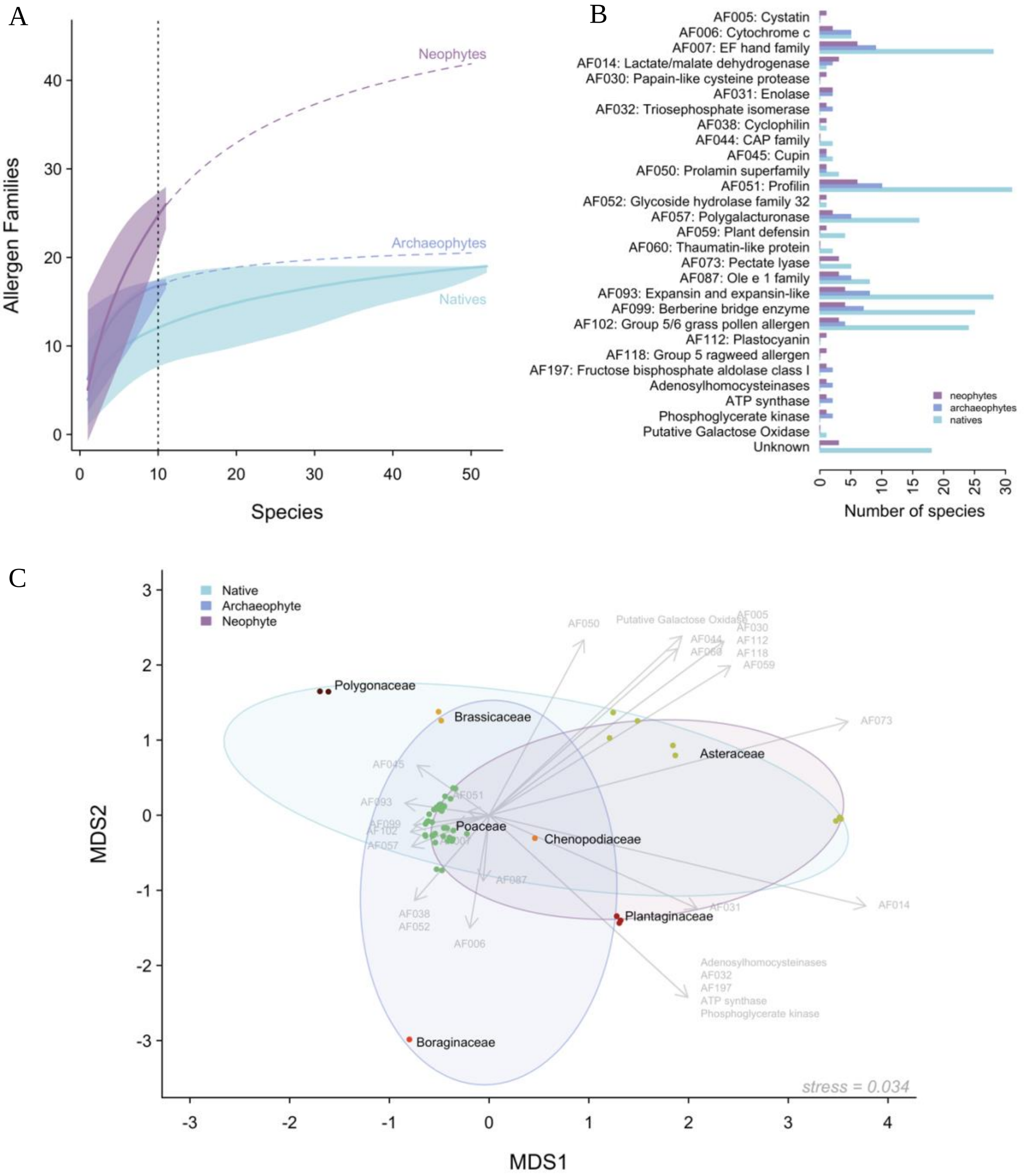

Figure S1. Distribution of allergen molecules and allergen protein families across species. A) Accumulation curves of allergen protein families with increasing species richness. Each solid curve was obtained by 10 000 random sampling of species within each pool of floristic status (bold lines: mean values; shaded areas: 95% confidence intervals), and was extrapolated by fitting a Lomolino regression (dashed curves). Neophyte species accumulate new allergen families much faster than natives or archaeophytes. The mean number of allergen families for 10 species (vertical dotted line) was: natives, 12.05 ± 2.79; archaeophytes, 16.82 ± 0.57; neophytes, 24.73 ± 2.18. **B)** Distribution of 29 allergen protein families across native, archaeophytes and neophyte species of the Berlin grasslands. The length of each bar corresponds to the number of species whose pollen contains at least one allergenic protein belonging to the protein family. Allergen families are identified by an AllFam code (e.g. "AF005"). Additional unclassified protein families, assigned to some allergen peptides in the literature, are listed at the bottom. **C)** Non metric multidimensional scaling of 51 allergenic species (dots) in allergen molecule family space (grey arrows)**.** Colours of dots represent the taxonomic family of allergenic plant species. Convex hull ellipses illustrate the three distribution of the three floristic groups (Native, Archaeophytes, Neophytes).

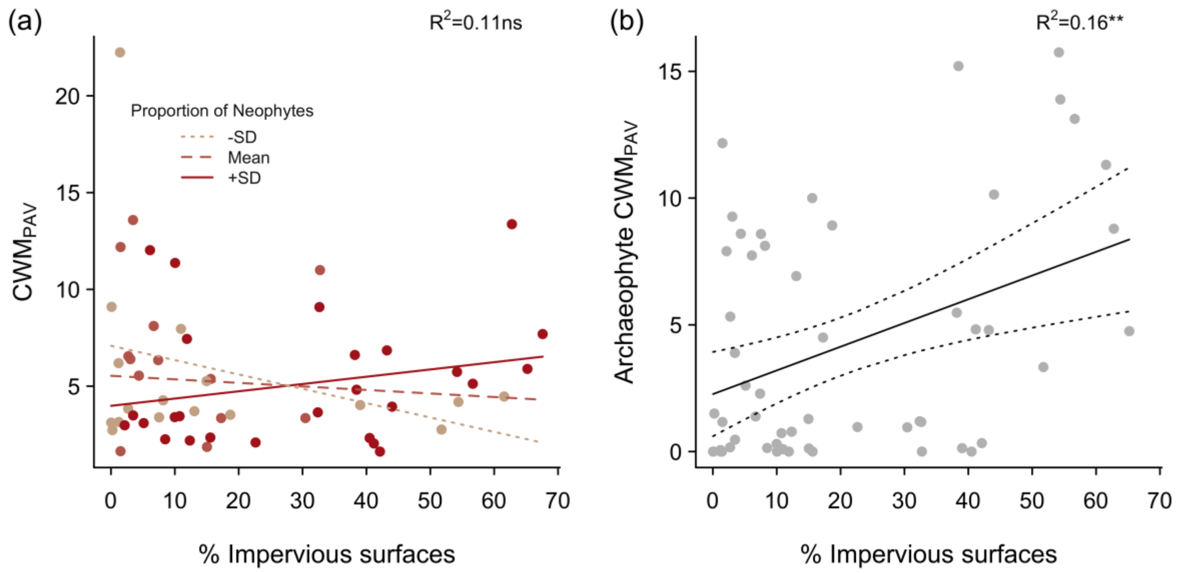

Figure S2. Abundance-weighted potential allergenic value (CWM_{PAV}) of grassland communities along the urbanisation gradient (% Impervious surfaces). (a) CWM_{PAV} tended to increase with urbanisation when the proportion of neophytes was high, although the model was not significant. This positive interaction is illustrated with partial regression lines for three values of proportion of neophytes (*-SD* = 0.016, *Mean* = 0.065, *+SD* = 0.113). Statistics for the non-significant linear model with interaction are indicated. (b) CWM_{PAV} of archaeophytes present in each community increased significantly with urbanisation. Statistics for the linear model are indicated (details in Table S3; ns, P > 0.05; **, P < 0.01).

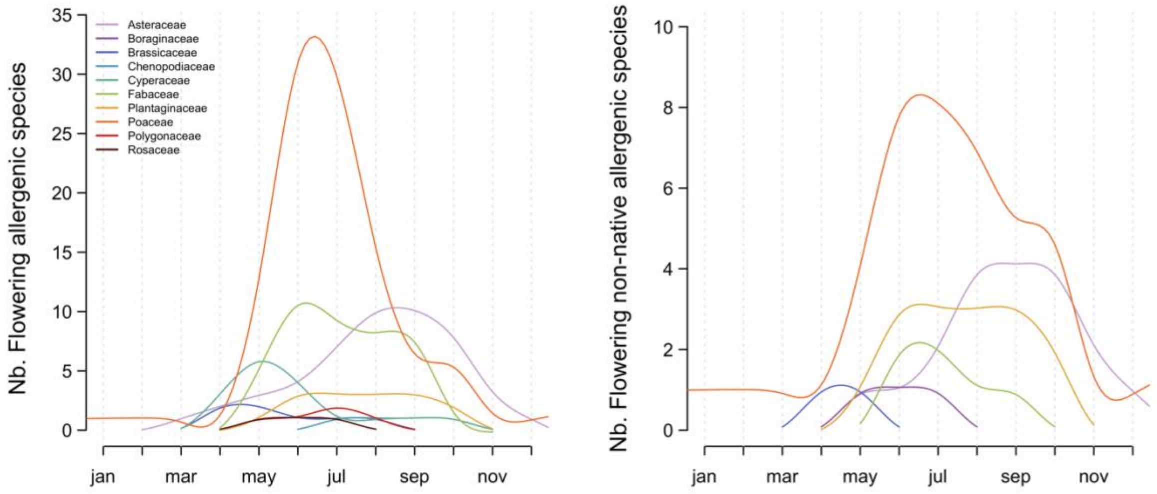

Figure S3. Flowering distribution of allergenic species grouped by taxonomic families. The number of flowering allergenic species per month in each family was determined based on species first and last month of flowering. Curves represent cubic smoothing splines fitted on the monthly data. The left-hand panel represents all 74 allergenic species in the Berlin grasslands, while the right-hand panel represents only the subset of non-native allergenic species.

Appendix S3: Collecting allergenicity data

Overview

We collected data on the allergenic properties of the 216 herbaceous plant species recorded along the 56 grassland plots using mainly three online allergen databases (Sircar et al. 2014) presented in **Table S3.1**. By restricting our search criteria to plant pollens, we obtained data on the identity of allergen peptides present in the pollen of plant species, sometimes accompanied by a scoring of allergenic severity, information on known symptoms, and prevalence in human populations (mostly from the northern hemisphere).

Table S3.1. Allergen databases used in this study. Number of matches correspond to the number of species or genus in the vegetation surveys which could be matched to a name in the database.

Database	Description	Matches at species level	Matches at genus level	Link
IUIS	Online database curated by the Allergen Nomenclature Sub-Committee of the WHO and the International Union of Immunological Societies (IUIS). Has the most stringent criteria.	10	25	http://www.allergen.org
FARRP	Downloadable allergen dataset evaluated by a peer-review panel of scientists and clinicians	9	23	http://www.allergenonline.org
Allergome	Most comprehensive online allergen database, including some allergens not fully supported by clinical tests. Provides, when available, the degree of allergenicity based on literature data.	21	39	http://www.allergome.org

Determining which species are allergenic

We first assembled evidence of pollen allergenicity for each of the species, in order to classify the species as allergenic or not. Any species listed in at least one of the databases was considered allergenic. Combining all three databases, which were only partially redundant, we were able to match 22 allergenic plant species from the Berlin vegetation survey dataset. We then added all grass species (*Poaceae* family) as allergenic, since they are well-known to produce major allergens (Esch et al. 2001; Andersson and Lidholm 2003; Weber 2015), which added 22 additional allergenic species. Finally, we checked the literature for additional primary studies concerning the remaining species, selecting only studies supported by publicly available clinical or immunological evidence of allergenicity (cf. dataset on species allergenicity). Applying this protocol, we identified 14 new allergenic species, which sums up to a total of 58 out of 216 species identified as allergenic.

However, this list of allergenic species was obviously limited by current knowledge in allergy research: the absence of a species in the database (or the literature) may be due either to a real absence of allergenic potential or a lack of research on this species. Often, only a single model species from a genus has been extensively studied (e.g. *Lolium perenne L.* or *Ambrosia artemisiifolia L.*). However, there is evidence of cross-reactivity of allergens within closely related species (Weber 2004; D'Amato et al. 2007), indicating that the more closely related two species are, the more likely they are to share

allergens. There are of course exceptions to this pattern, though mostly beyond the genus level (Weber 2007). Moreover, some taxonomic groups are known to be generally allergenic and cross reactive, such as grasses (Andersson and Lidholm 2003; Weber 2007). Therefore, we expanded the definition of allergenicity to include all congeneric species and all grasses, which raised the total number of allergenic species to 74. We believe this to be a reasonable correction to the negative bias created by publication gaps.

Assigning allergen molecules to allergenic species

We assembled information on known allergen molecules (hereafter allergens) present in the pollen of each allergenic species from the above-mentioned database. One species often produces multiple different allergens at the same time (e.g. 7 different molecules identified for *Artemisia vulgaris L.*). Allergen databases provide not only the name of allergens, but also the protein family to which they belong. Pollen allergens are peptides usually belonging to one of 29 main protein families (Radauer and Breiteneder 2006), although some allergens still remain unclassified (14/233 inhaled plant allergens remained unclassified on March 27th 2020 in the AllFam database). Allergen names follow a standardized nomenclature (Pomés et al. 2018) which combines the species name (e.g. "Art v" for *Artemisia vulgaris*) and a number which identifies the allergen among other allergens from the same species and often corresponds to a distinct biochemical group (i.e. protein family) within the family or genus (e.g. "Art v 1" is in the *Plant Defensin* family).

We complemented information on protein families by consulting the SDAP (Structural Database of Allergenic Proteins, https://fermi.utmb.edu/, curated by the University of Texas, USA), the AllFam database (http://urbanis.meduniwien.ac.at/allfam/browse.php, curated by the Medical University of Vienna, Austria), and additional primary literature when necessary (cf. references in the dataset). To simplify the data extracted from the databases, we grouped isoallergens, i.e. similar allergen molecules isolated by different studies or in different closely related taxa, under one simplified molecule name mimicking the WHO/IUIS nomenclature (Pomés et al. 2018). This data gathering provided records for 97 unique allergen molecules, belonging to 23 out of 74 of the allergenic species. Given the strict methodological requirements for registering new allergen molecules, gaps in published data are even larger for identifying allergen molecules than for allergen species. We attempted to fill this gap in knowledge based on known patterns of cross-reactivity between plant species in two ways: (1) by extrapolating molecules at the genus level and (2) by considering the special case of the grass family. These extrapolations (detailed below) allowed us to assign 122 unique allergens to 53 out of 74 allergenic species.

First, we assigned allergens at the genus level based on known allergens at the species level, given the well documented cross-reactivity within genera (Weber 2007). To avoid artificially inflating the number of different allergens, we re-named and merged all allergens at the genus level. For instance, as *Artemisia campestris L.* is a congeneric of *Artemisia vulgaris*, the allergen "Art v 1" became coded as the allergen "Artemisia_1" for both species. Results for this broad genus-level classification of allergen molecules were compared to results using the more conservative species-level definition (**Figure S3.1**) and found to follow similar trends. We thus decided to present only the more comprehensive results at the genus level.

Second, we assigned common allergens across grass species (i.e. members of the *Poaceae* family).For grasses, clear patterns of cross-reactivity have been well documented (Esch et al. 2001; Andersson and Lidholm 2003; Weber 2004), meaning that grasses tend to share similar allergen molecules, at least at the sub-family or tribe level. Twelve groups of grass pollen allergens have been identified, of which three (Group 1, 4 and 5) are particularly widespread. Accordingly, Group 1 (*AF093: Expansin and expansin-like*) and Group 4 (*AF099: Berberine bridge enzyme*) grass allergens were attributed to all *Poaceae* species, and Group 5 (*AF102: Group 5/6 grass pollen allergen*) allergens were attributed to grasses from the *Pooideae* subfamily. Cross-reactivity also means that assigning these molecules at the species level would artificially inflate the number of apparently different allergen molecules. To avoid such an inflation, we assigned molecule names from these three groups at the tribe level where cross-reactivity is clearest (Weber 2003; e.g. *"Poe 5"* was the common allergen name of Group 5 for all grasses from the *Poe* tribe). Names of known grass allergens from other allergen groups were kept unchanged (i.e. at the genus level).

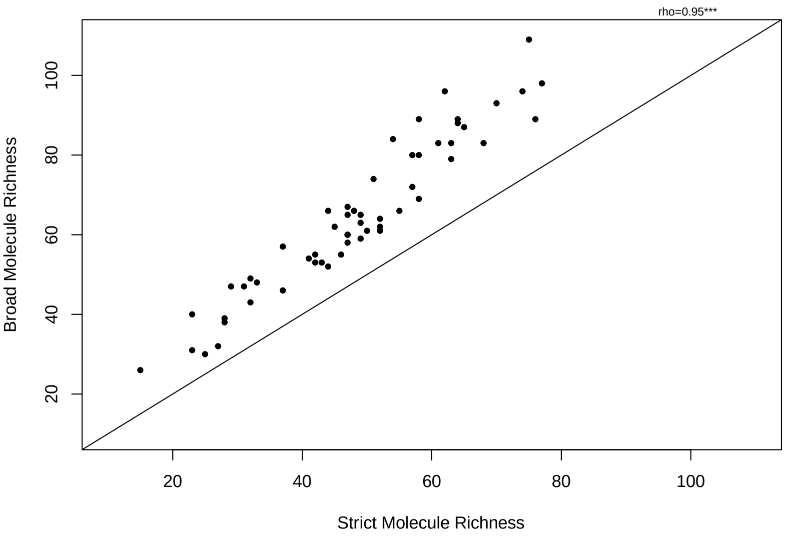

Figure S3.1. Comparing allergen molecule richness calculated with strict vs. broad attributions of allergen molecules. In the strict version, allergen molecules were assigned at the species level; in the broad definition, at the genus level (cf. above). Molecule richness was calculated as the total number of unique molecules present in each grassland site. The black line represents the identity line (1:1). Statistics for Spearman's rank correlation are indicated (*** : P < 0.001)

Calculating species potential allergenic values

We calculated the potential allergenic value (PAV) for each species following the method proposed by Cariñanos and collaborators (Cariñanos et al. 2014). We assigned an overall allergenic potential score to species by multiplying three main factors: allergenicity score (from 0 to 4; details below and **Table S3.2**); pollination syndrome (four categories: 0-no pollen emissions, 1-biotic pollination or low emissions, 2-mixed pollination with moderate pollen emissions, and 3-wind-pollinated with high pollen emissions); and duration of pollination (scores from 1 to 3: <1 month, <2 months, and > 2 months). These ordinal scores were multiplied, establishing an overall score ranging from 0 to 36 for each species.

To calculate pollination syndrome scores, we used information on pollination syndromes extracted from the *BiolFlor* database. Note that in Cariñanos et al. (2014), the categories for duration of pollination were slightly different and expressed in weeks: 3 weeks, 6 weeks, and >6 weeks. As our phenological data was resolved only to the month, we had to adjust the categories to cover whole months.

Table S3.2. Allergenicity scale used to score each species. The different criteria used to assign a score to each of the 216 species in the Berlin grasslands are listed in the description. For scores 2-4, at least one of the criteria had to be met.

Allergenicity Score	Description
**No known allergenicity** (0)	No evidence of allergenicity; No known cross-reactivity within the genus or other taxonomic levels.
**Mild** (1)	Low allergenicity, with low reactivity (<5%) in hay fever patients (e.g. skin prick tests, nasal provocations). Low cross reactivity with other allergen sources.
**Moderate** (2)	Intermediate reactivity; moderate reactivity (5-20%) in hay fever patients; Possible partial cross-reactivity with major allergens of hay fever.
**High** (3)	High allergenicity; a high prevalence of reactivity (20-60%) in hay fever patients; High cross-reactivity with major allergens of hay fever.
**Very high** (4)	Identified as a major allergen; very high prevalence (> 60%) in hay fever patients

Allergenicity scores were assigned for each species based on clinical or immunological information provided in the allergen databases regarding severity or reactivity prevalence in patients suffering from hay fever, complemented when necessary with additional information from the primary scientific literature (sources and rationale for each species are provided in the online dataset). We followed a five-point scoring scale (**Table S3.2**) modelled after the one proposed by Cariñanos et al. (2014). The highest category ("very high allergenicity", score = 4), which in Cariñanos *et al.* (2014) corresponded to major allergens of the Mediterranean region, was modified to include all recognized major allergens in Europe. Only *Artemisia vulgaris L., Ambrosia psilostachya* DC. and those grass species recognized by the IUIS as major allergens (e.g. *Lolium perenne L.*) were assigned a score of 4. Once again, we generalized allergenic scores at the genus level, except for the few species with a score of 4, which we considered to be notable major allergens even among their congeners.

References

Andersson, K., and J. Lidholm. 2003. Characteristics and immunobiology of grass pollen allergens. *International Archives of Allergy and Immunology* 130: 87–107. doi:10.1159/000069013.

Cariñanos, P., M. Casares-Porcel, and J.-M. M. Quesada-Rubio. 2014. Estimating the allergenic potential of urban green spaces: A case-study in Granada, Spain. *Landscape and Urban Planning* 123. Elsevier: 134–144. doi:10.1016/j.landurbplan.2013.12.009.

D'Amato, G., L. Cecchi, S. Bonini, C. Nunes, I. Annesi-Maesano, H. Behrendt, G. Liccardi, T. Popov, et al. 2007. Allergenic pollen and pollen allergy in Europe. *Allergy: European Journal of Allergy and Clinical Immunology* 62. Wiley/Blackwell (10.1111): 976–990. doi:10.1111/j.1398-9995.2007.01393.x.

Esch, R. E., C. J. Hartsell, R. Crenshaw, and R. S. Jacobson. 2001. Common allergenic pollens, fungi, animals, and arthropods. *Clinical Reviews in Allergy and Immunology* 21: 261–292. doi:10.1385/CRIAI:21:2-3:261.

Pomés, A., J. M. Davies, G. Gadermaier, C. Hilger, T. Holzhauser, J. Lidholm, A. L. Lopata, G. A. Mueller, et al. 2018. WHO/IUIS Allergen Nomenclature: Providing a common language. *Molecular Immunology* 100: 3–13. doi:10.1016/j.molimm.2018.03.003.

Radauer, C., and H. Breiteneder. 2006. Pollen allergens are restricted to few protein families and show distinct patterns of species distribution. *Journal of Allergy and Clinical Immunology* 117: 141–147. doi:10.1016/j.jaci.2005.09.010.

Sircar, G., D. Sarkar, S. G. Bhattacharya, and S. Saha. 2014. Allergen Databases. In *Immunoinformatics*, ed. Rajat K. De & Namrata Tomar, 1184:165–181. New York, NY: Springer New York. doi:10.1007/978-1-4939-1115-8_9.

Weber, R. W. 2003. Patterns of pollen cross-allergenicity. *Journal of Allergy and Clinical Immunology* 112: 229–239. doi:10.1067/mai.2003.1683.

Weber, R. W. 2004. Cross-reactivity of pollen allergens. *Current Allergy and Asthma Reports* 4: 401–408. doi:10.1007/s11882-004-0091-4.

Weber, R. W. 2007. Cross-reactivity of pollen allergens: Impact on allergen immunotherapy. *Annals of Allergy, Asthma and Immunology* 99. American College of Allergy, Asthma & Immunology: 203–212. doi:10.1016/S1081-1206(10)60654-

0.
Weber, R. W. 2015. Outdoor Allergens. In *Pediatric Allergy: Principles and Practice: Third Edition*, Third Edit, 185–185. Elsevier Inc. doi:10.1016/B978-0-323-29875-9.00020-3.

Appendix S4: Turnover in allergen composition

Using distance-based redundancy analyses, we quantified trends in dissimilarities (Jaccard) between grassland communities in terms of allergen species composition as well as allergen molecule and allergen protein family composition (**Figure S4.1**). We found a significant turnover in the composition of allergen species, molecules and families with urbanisation (% Impervious surfaces). The proportion of neophytes was associated to a turnover only in allergen molecule and allergen protein family composition (**Figure S4.1b,c**). These patterns of turnover had a relatively low effect size (low partial R^2), indicating other sources of variation in composition which we did not capture. Models remained consistent when considering species local abundances (dbRDA with Bray-Curtis dissimilarities; not shown).

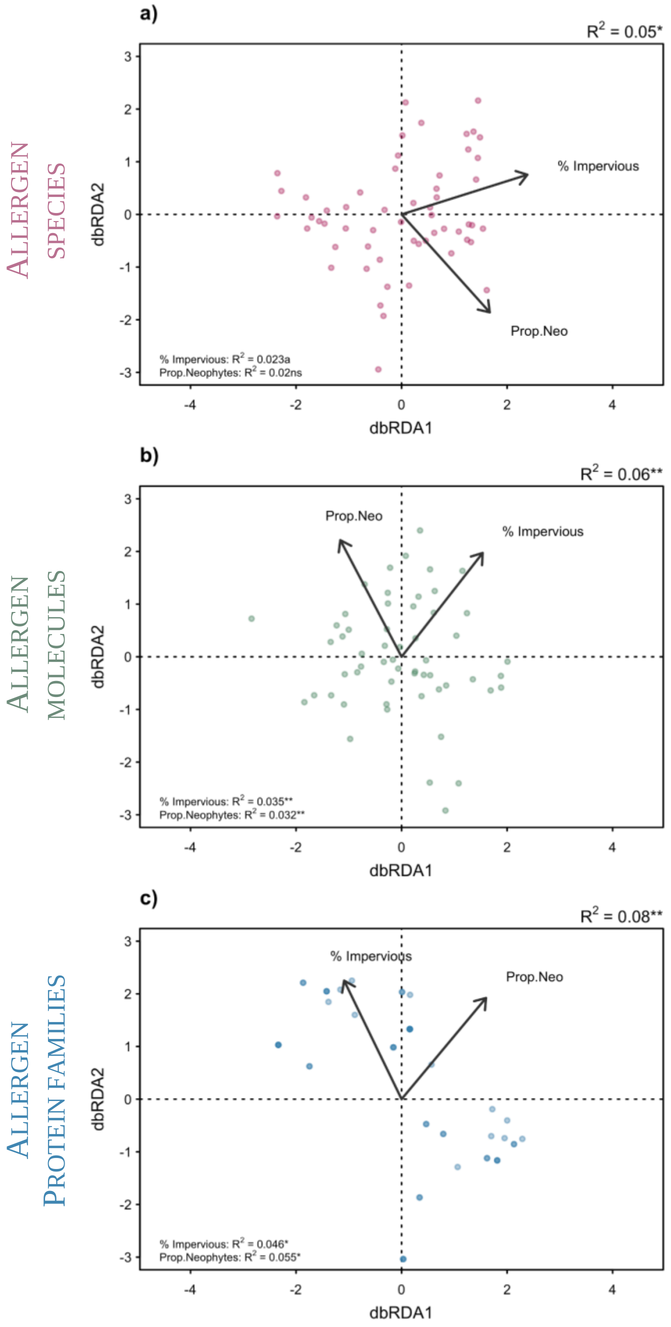

Figure S4.1. Turnover in composition of allergenic species (a), allergen molecules (b) and allergen protein families (c) in Berlin grasslands as a function of urbanisation and diversity. We present the first two axes of distance-based redundancy analyses (dbRDA) calculated using Jaccard distances between grassland communities (total R2 and pseudo-F tests in the upper right corners; 999 permutations). Analyses were carried out with two predictors: species % Impervious surfaces in 500m (% Impervious) and % of neophyte species (Prop.Neo). Predictor scores (arrows) and statistics (partial marginal R2) are shown for the two parameters which were tested as constraints in the dbRDAs. Each point represents a grassland site. (**, $P < 0.01$; *, $P < 0.05$; a, $P < 0.1$; ns, $P \geq 0.05$).
